# Is the Cell Nucleus a Necessary Component in Precise Temporal Patterning?

**DOI:** 10.1371/journal.pone.0134239

**Published:** 2015-07-30

**Authors:** Jaroslav Albert, Marianne Rooman

**Affiliations:** 1 BioModeling, BioInformatics & BioProcesses, Université Libre de Bruxelles, Brussels, Belgium; 2 Applied Physics Research Group, Vrije Universiteit Brussel, Brussels, Belgium; Imperial College London, UNITED KINGDOM

## Abstract

One of the functions of the cell nucleus is to help regulate gene expression by controlling molecular traffic across the nuclear envelope. Here we investigate, via stochastic simulation, what effects, if any, does segregation of a system into the nuclear and cytoplasmic compartments have on the stochastic properties of a motif with a negative feedback. One of the effects of the nuclear barrier is to delay the nuclear protein concentration, allowing it to behave in a switch-like manner. We found that this delay, defined as the time for the nuclear protein concentration to reach a certain threshold, has an extremely narrow distribution. To show this, we considered two models. In the first one, the proteins could diffuse freely from cytoplasm to nucleus (simple model); and in the second one, the proteins required assistance from a special class of proteins called importins. For each model, we generated fifty parameter sets, chosen such that the temporal profiles they effectuated were very similar, and whose average threshold time was approximately 150 minutes. The standard deviation of the threshold times computed over one hundred realizations were found to be between 1.8 and 7.16 minutes across both models. To see whether a genetic motif in a prokaryotic cell can achieve this degree of precision, we also simulated five variations on the coherent feed-forward motif (CFFM), three of which contained a negative feedback. We found that the performance of these motifs was nowhere near as impressive as the one found in the eukaryotic cell; the best standard deviation was 6.6 minutes. We argue that the significance of these results, the fact and necessity of spatio-temporal precision in the developmental stages of eukaryotes, and the absence of such a precision in prokaryotes, all suggest that the nucleus has evolved, in part, under the selective pressure to achieve highly predictable phenotypes.

## Introduction

Biological cells house an enormous number of diverse molecules that interact with the cells’ environment and among each other. Such a complex network of interactions makes the evolution of any molecule stochastic, be it the change in its position, conformation, or copy number. The effects stochasticity has on the phenotype of a cell are many, ranging from significant to negligible; in some cases they are detrimental [1], in others they play an important role [2]. For example, a population of *Escherichia coli* may survive the attack from an antibiotic drug thanks to random switching between two phenotypes—one sensitive, the other resistant to the drug [3]. Sporulation in yeast is another example; in this case the concentration level of a single protein determines the fate (death or survival) of a cell [4]. In eukaryotes, too, one can observe noise-induced subpopulations at work, such as was done for the immune T-cells [5]. Even in the early development of eukaryotes, where spatio-temporal precision is essential, stochastic variations can be of service, as was exemplified by the segmentation clock [6]: the oscillating expression of the gene *her1* is sustained by virtue of copy number fluctuations.

As essential as these mechanisms may appear, observation, experimentation and modeling of gene expression suggest that important genes must be (and are) tightly regulated [7–9]. And in fact, numerous mechanisms of noise reduction and noise filtering have been discovered. Negative transcriptional [10, 11] and translational [8, 12] autoregulation, non-linear protein degradation [13], multimer formation [14–16] are just a few examples worth mentioning here. Especially in the developmental stages of eukaryotes is noise reduction desirable: the spatio-temporal execution of the genetic program must be as noise free as possible [17, 18].

Previously, studies on the regulation of noise in development have focused mainly on stabilization of newly differentiated cell states [17], noise filtering of morphogen gradients [18], how cellular boundaries are formed and maintained [19], and how the segmentation clock, e. g. in Zebrafish, can lead to synchronized oscillations due to cell signaling [20, 21]. In this paper, we investigate how the segregation of the nuclear space from that of the cytoplasm affects the timing accuracy of gene expression. The effects of such division have been investigated in previous studies, e. g. in the context of Notch signaling and synchronized oscillations [20], the Hes1 and p53-Mdm2 pathways [22], and how the intrinsic noise, coupled with the effects of the nuclear membrane, can explain heterogeneity in embryonic stem cell differentiation [23]. Motifs with autoregulation have also been studied with emphasis on the spatial degree of freedom [24]. While all these studies do incorporate the presence of the nuclear membrane, they do not focus specifically on the question of whether such separation can lead to enhanced timing accuracy in gene transcription. It is this niche that we try to fill in this paper.

What we consider are two models of protein transport from cytoplasm to nucleus. In the first model, we allow the protein to diffuse freely across the nuclear envelope. The second model involves a three-gene motif comprising of the gene of interest and two other genes that code for importin-*α* and importin-*β*—two out of a class of proteins responsible for facilitating nuclear import of other proteins. Both models include a negative feedback. The most simplistic version of this motif, one which does not involve the nuclear envelope and hence importins, has been extensively studied and is well understood: the time for a gene to reach a steady state is fast and the noise diminished compared to the situation with no negative feedback. In order to prolong the time to reach a steady state, a positive feedback has been the traditional device; however, this leads to an increase of noise. We demonstrate that the inclusion of the cell nucleus can also lead to a prolonged transition into a steady state, however, at the same time, to a reduction of noise—the very opposite of the nucleus-free situation.

Furthermore, we explore the possibility that without the nucleus it is not possible to generate a delayed transition while also reducing the noise. We do this by comparing the coherent feed-forward motif (CFFM), and variations thereof, to the motif involving a nucleus. The CFFM is highly abundant in many prokaryotes [25], which makes it a good first step. Our results show that although it is possible to match the temporal profile of the protein concentration inside the nucleus with that of the protein coded for by the third and last gene downstream of the CFFM, the noise in the latter will tend to be much greater during the transition into a steady state. In light of these results, we dare ask whether the cell nucleus is an indispensable component of the near deterministic workings of the developmental gene regulatory networks in eukaryotes.

## Results

### Modeling mRNA and protein concentration in eukaryotes

**Fig 1:**
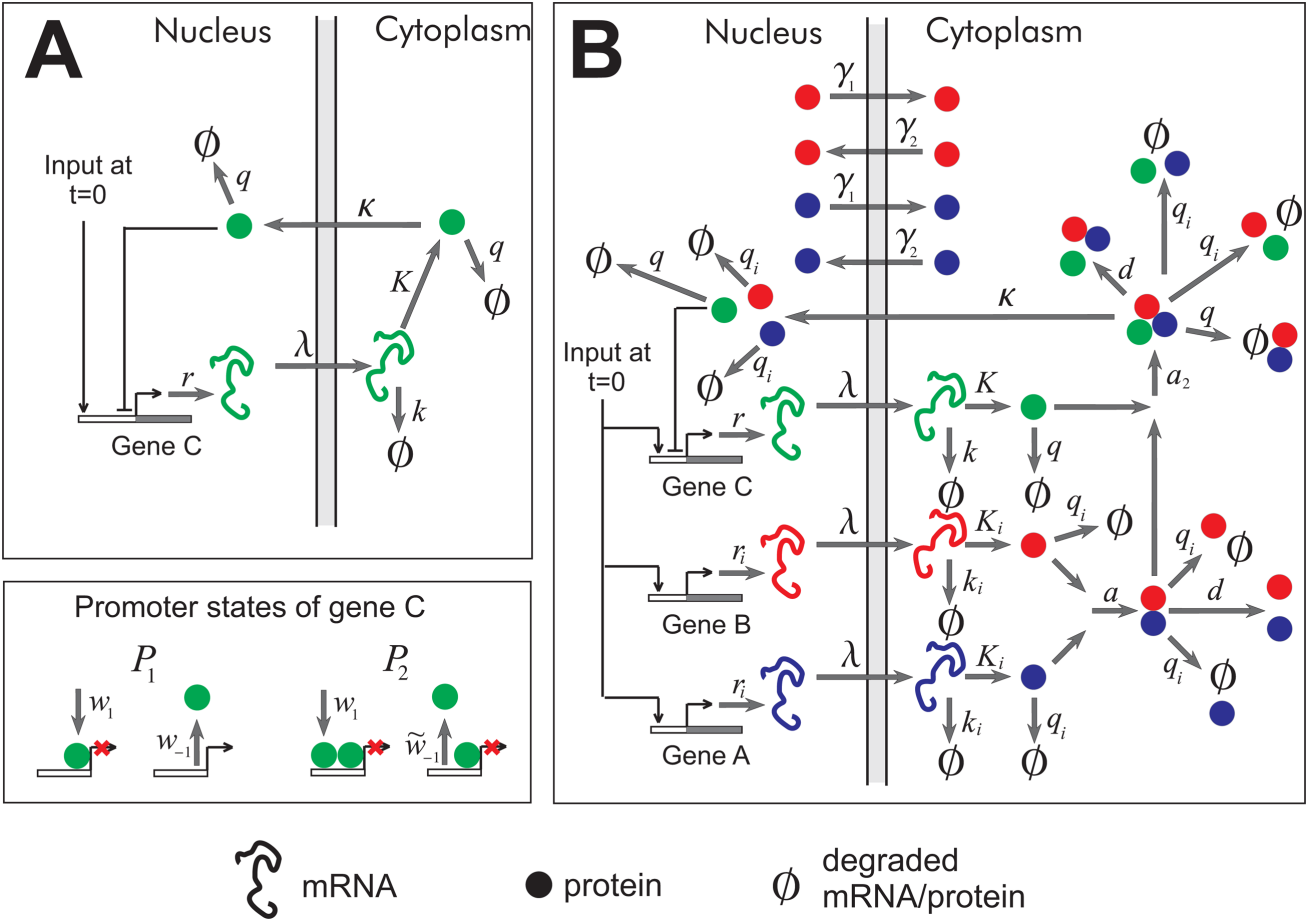
Two models of nuclear transport. A) Simple model: the protein of gene C diffuses freely from cytoplasm into the nucleus. B) Extended model: a gene network involving importin-*α* (blue), importin-*β* (red) and cargo protein (green). The symbol ∅ indicates degraded protein and mRNA. The reactions are labeled as in Eqs (3, 2, 5, 6): *r*—transcription rate; *λ*—export rate of mRNA into the cytoplasm; *K*—translation rate; *k*—rate of mRNA degradation; *q*—rate of protein degradation; *a* (*d*)—rate of association (dissociation) between importins *α* and *β*; *a*_2_ (*d*)—rate of association (dissociation) between the importin-*α*−*β* complex and cargo protein; *κ*—import rate of the importin-*α*−*β*-cargo complex; *γ*_1_—rate export of importins *α* and *β* into cytoplasm; *w*_1_ (*w*_−1_)—rate of association (dissociation) between a cargo protein and a free promoter of gene C; ${\tilde{w}}_{-1}$—rate of dissociation of a cargo protein from a fully occupied promoter.

For an mRNA molecule to be translated, it must find its way from the nucleus into the cytoplasm. The mRNA recruits special proteins with which it forms a complex [26] that freely diffuses into the cytoplasm [27]. Once in the cytoplasm, the mRNA rarely returns back to the nucleus, and so, from now on we will consider its transport a one way trip. Also, it is only in the cytoplasm that mRNA is actively degraded; in the nucleus, only damaged mRNA is degraded. The mechanism of intra-compartmental locomotion of proteins depends (in part) on their size: small proteins (< ∼ 40kDa) tend to diffuse freely across the membrane, while large ones need assistance from a family of proteins known as importins and exportins [28]. Those proteins that are needed back in the nucleus after translation, e.g. transcription factors, contain an amino acid sequence that the importins recognize and bind. The simplest scenario involves only one type of importin, importin-*β*. In another, more complex, situation two importins, importin-*α* and importin-*β*, work together to facilitate transport of cargo proteins across the nuclear membrane. In the most likely scenario, importin-*α* binds to importin-*β* before it can bind to the cargo protein. Only in this three-protein complex is import into the nucleus most efficient—though the importin-*β*–cargo complex, too, can sneak in but with a significantly smaller rate. Fig 1 shows in detail the essential reactions involved in synthesizing and transporting mRNA and protein. Models of this type of system have been studied previously, both theoretically and experimentally [28–30]

In the next two sections, we set up the deterministic equations for both the free diffusion (simple) and the importin-assisted (extended) models, and in the section after that we analyze the stochastic dynamics of these two models.

#### Deterministic model—simple

**Fig 2:**
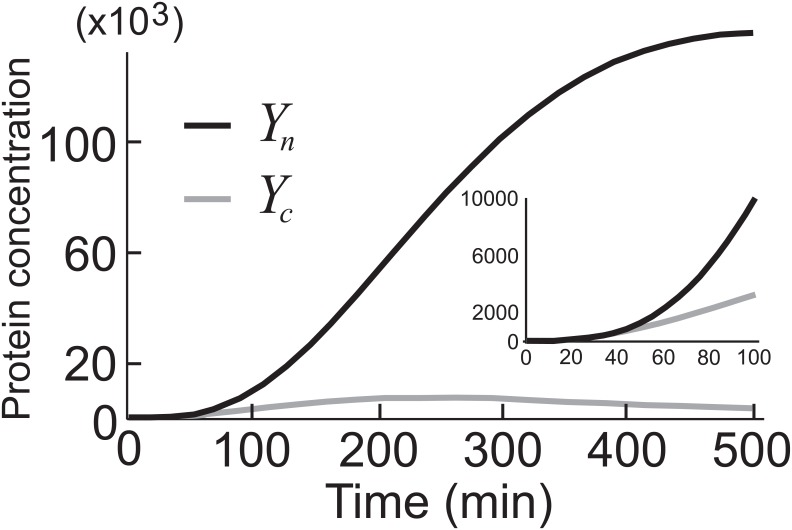
Evolution of average protein concentrations. A close-up view of the figure is given on the right-hand side. The nuclear and the cytoplasmic concentrations rise at approximately the same rate until *t* ≈ 40, after which time the nuclear concentration begins to increase much more rapidly.

We model the eukaryotic transcriptional and translational machinery in a two-compartment cell: a negatively auto-regulated gene is transcribed in the nucleus; the generated mRNA diffuses through the membrane from the nucleus to the cytoplasm, where it is translated into protein; the protein then diffuses freely into the nucleus. Let us write down the set of equations governing the dynamics of mRNA and protein concentrations. We denote the four essential processes, the rate of transcription, translation, and mRNA and protein degradation, as *r*, *K*, *k* and *q* respectively. Then, the concentrations of mRNA, *X*_*n*_, and protein, *Y*_*n*_, inside the nucleus, and of the mRNA, *X*_*c*_, and protein, *Y*_*c*_, in the cytoplasm obey:

$$\begin{array}{ccc} & & {\dot{X}}_{n}=r\left(1-P_{1}-P_{2}\right)-\lambda X_{n},\\ & & {\dot{X}}_{c}=\lambda X_{n}-kX_{c},\\ & & {\dot{Y}}_{n}=\kappa Y_{c}-qY_{n},\\ & & {\dot{Y}}_{c}=KX_{c}-\left(q+\kappa \right)Y_{c}, \end{array}$$ (1)

where *λ* is the rate of mRNA diffusion into the cytoplasm, while *κ* is the rate of protein diffusion from the cytoplasm into the nucleus. The promoter states *P*_1_ and *P*_2_ are governed by:

$$\begin{array}{ccc} {\dot{P}}_{1} & = & w_{1}Y_{n}\left(1-P_{1}-P_{2}\right)+{\tilde{w}}_{-1}P_{2}-w_{-1}P_{1}-w_{1}Y_{n}P_{1}\\ {\dot{P}}_{2} & = & w_{1}Y_{n}P_{1}-{\tilde{w}}_{-1}P_{2}, \end{array}$$ (2)

where *w*_1_ and *w*_−1_ are the rates at which *Y*_*n*_ binds and dissociates a free promoter; ${\tilde{w}}_{-1}$ is the rate at which *Y*_*n*_ decouples from a fully occupied promoter *P*_2_. In Fig 2, we show the dynamics of the variables *Y*_*n*_ and *Y*_*c*_ for typical parameters.

#### Deterministic model—extended

Let us now consider the role of importins in the transport of cargo proteins. Before proceeding, we make the following assumptions:

- mRNA does not degrade inside the nucleus;
- mRNA does not diffuse back into the nucleus;
- The rate of diffusion into the cytoplasm is the same for all species of mRNA λ;
- There is no export of cargo proteins from the nucleus to the cytoplasm;
- Importins *α* and *β* diffuse freely in and out of the nucleus;
- The rates of transcription (*r_i_*), translation (*K_i_*), mRNA degradation (*k_i_*), and protein degradation (*q_i_*) associated with importin-*α*, are identical to those associated with importin-*β*;
- The rate of dissociation *b* of importin-*α* from importin-*β* is equivalent to that at which the importin-*α*-importin-*β* complex dissociates from the cargo protein;
- Proteins in the nucleus degrade at the same rate as those in the cytoplasm;
- Degradation rates for all species of protein remain constant under multimerization;
- The promoter occupancy of gene C, i.e. *P*_1_ and *P*_2_ in Fig 1B, corresponding to one and two proteins bound to it respectively, occurs with the same rate (*w*_1_).
- The concentrations of all gene products are zero at t = 0.

All these assumptions are true to various degrees for various gene products in the real world. Our main reason for making them is to reduce the number of parameters, which, we argue, does not diminish the significance of our results: whatever this system can achieve with fewer parameters, it will necessarily achieve with more parameters. The last assumption may be an oversimplification, as the regulation of both importin-*α* and importin-*β* is more complicated by virtue of being part of a larger gene network [31–33]; same applies to the cargo protein. However, for our purposes here, which are to investigate the role of the cell nucleus in concentration dynamics and in the regulation of intrinsic noise, it will suffice as the first step. Discussion of the situation where the importins start out with a non-zero concentration, e. g. in a steady state, will follow in the “Discussion” section.

Let us write down the set of equations governing the dynamics of mRNA and protein concentrations under the above mentioned assumptions. Using the same notation for the cytoplasmic and nuclear mRNA and cargo protein as in the previous section, the equations of motion read:

$$\begin{array}{ccc} & & {\dot{X}}_{n}=r\left(1-P_{1}-P_{2}\right)-\lambda X_{n},\\ & & {\dot{X}}_{c}=\lambda X_{n}-kX_{c},\\ & & {\dot{Y}}_{n}=\kappa Y_{c}-qY_{n},\\ & & {\dot{Y}}_{c}=KX_{c}+\left(d+2q_{i}\right)Z_{3}-a_{2}Z_{2}Y_{c}-qY_{c}, \end{array}$$ (3)

where *Z*_2_ is the importin-*α*–importin-*β* heterodimer, and *Z*_3_ is the importin-*α*–importin-*β*–cargo complex. The coefficient *a*_2_ is the binding rate of *Y*_*c*_ to *Z*_2_, and *d* the rate at which they dissociate. As before, *κ* is the transport rate into the nucleus, but this time it is *Z*_3_ that is being transported. Lastly, *q*_*i*_ stands for the degradation rate of both types of importins.

$$\begin{array}{ccc} {\dot{P}}_{1} & = & w_{1}Y_{n}\left(1-P_{1}-P_{2}\right)+{\tilde{w}}_{-1}P_{2}-w_{-1}P_{1}-w_{1}Y_{n}P_{1}\\ {\dot{P}}_{2} & = & w_{1}Y_{n}P_{1}-{\tilde{w}}_{-1}P_{2}, \end{array}$$ (4)

The equations for the *α*- and *β*-importins in the cytoplasm and nucleus *Y*_*αn*_, *Y*_*αc*_, *Y*_*βn*_, *Y*_*βc*_ and the corresponding mRNAs, *X*_*αn*_, *X*_*αc*_, *X*_*βn*_, *X*_*βc*_ from which they are translated, read:

$$\begin{array}{ccc} & & {\dot{X}}_{\alpha n}=r_{i}-\lambda X_{\alpha n},\\ & & {\dot{X}}_{\alpha c}=\lambda X_{\alpha n}-k_{i}X_{\alpha c},\\ & & {\dot{X}}_{\beta n}=r_{i}-\lambda X_{\beta n},\\ & & {\dot{X}}_{\beta c}=\lambda X_{\beta n}-k_{i}X_{\beta c},\\ & & {\dot{Y}}_{\alpha n}=\gamma_{2}Y_{\alpha c}-\gamma_{1}Y_{\alpha n}+\kappa Z_{3}-q_{i}Y_{\alpha n}\\ & & {\dot{Y}}_{\alpha c}=-\gamma_{2}Y_{\alpha c}+\gamma_{1}Y_{\alpha n}+K_{i}X_{\alpha c}+\left(d+q_{i}\right)Z_{2}+q_{i}Z_{2}-aY_{\alpha c}Y_{\beta c}-q_{i}Y_{\alpha c}\\ & & {\dot{Y}}_{\beta n}=\gamma_{2}Y_{\beta c}-\gamma_{1}Y_{\beta n}+\kappa Z_{3}-q_{i}Y_{\beta n}\\ & & {\dot{Y}}_{\beta c}=-\gamma_{2}Y_{\beta c}+\gamma_{1}Y_{\beta n}+K_{i}X_{\beta c}+\left(d+q_{i}\right)Z_{2}+q_{i}Z_{2}-aY_{\alpha c}Y_{\beta c}-q_{i}Y_{\beta c}. \end{array}$$ (5)

The coefficients *γ*_1_ = *γ* and *γ*_2_ = *νγ* are the free diffusion rates for importins, with *ν* = *V*_*n*_/*V*_*c*_ being the ratio of the volume of the nucleus to the volume of the cytoplasm. For the protein complexes, we have

$$\begin{array}{ccc} & & {\dot{Z}}_{2}=aY_{\beta c}Y_{\alpha c}-\left(d+2q_{i}\right)Z_{2},\\ & & \dot{Z_{3}}=a_{2}Z_{2}Y_{c}-\left(d+2q_{i}+q+\kappa \right)Z_{3}. \end{array}$$ (6)

The importin-*α*- importin-*β* heterodimer binds to (dissociates from) *Y*_*c*_ at the rate of *a*_2_ (*d*).

Let us now select values for all the parameters and solve numerically Eqs (3), (5) and (6). Fig 2 shows the temporal profiles of all proteins and their complexes in a) cytoplasm and b) the nucleus. All three genes are turned on at *t* = 0; this may be the result of a signaling molecule or the unraveling of the chromatin(s) harboring these genes. The most relevant feature for our purposes is the delayed production of *Y*_*n*_. For the first forty minutes or so its concentration remains low, and then begins rapidly increasing. This switch-like behavior is common in many biological systems. In what follows, we look at the stochastic behavior of this system and how the delays in the production of *Y*_*n*_ change from cell to cell; stated otherwise, we analyze the noise.

#### Stochastic model

**Fig 3:**
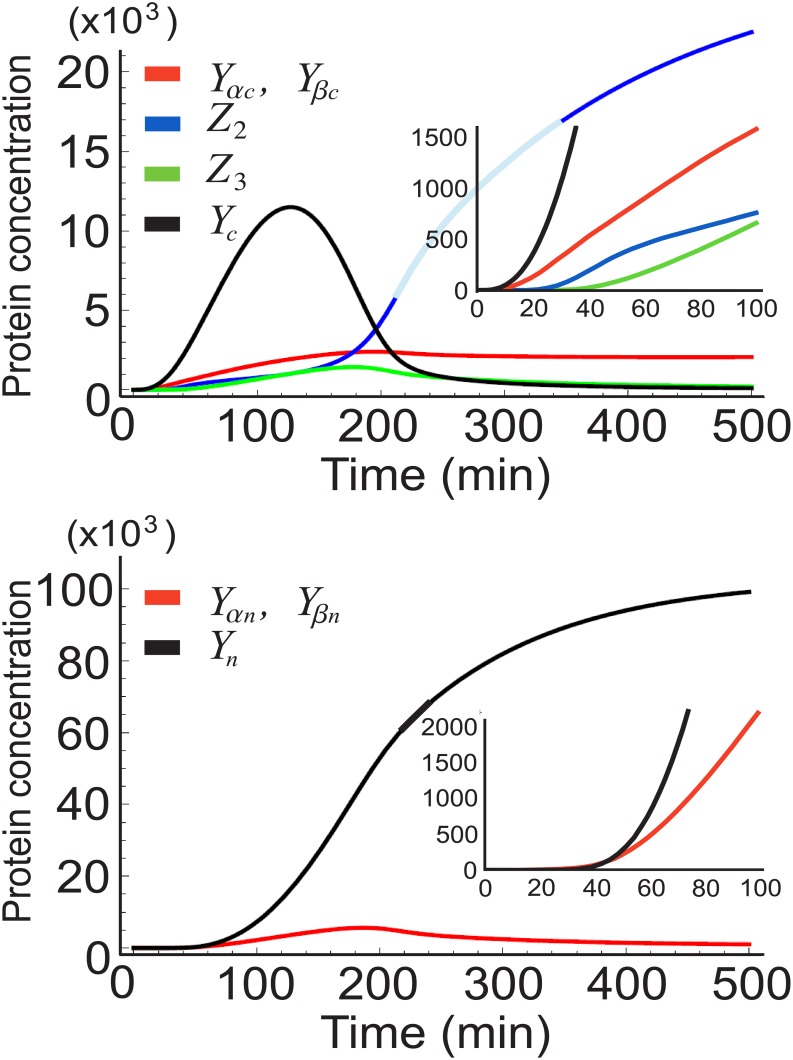
Evolution of average protein concentrations. A close-up view of the figure is given on the right-hand side A) Protein concentrations in the cytoplasm. At t = 0 all three genes are turned on. The cargo protein in the cytoplasm (black) begins to accumulate first, followed by the importin *α* and *β* (red). At about t = 20 min, importins *α* and *β* start forming heterodimers (blue), and 20 min after that, the concentration of importin-*α*−*β*-cargo complex (green) begins to rise. B) Protein concentrations in the nucleus. Both the cargo protein (black) and the importins *α* and *β* (red) start to accumulate at about t = 40 min.

Our goal in this section is not only to look at noise in the system defined by the parameters in Figs 2 and 3, but also to study how much the noise changes when we change these parameters. We will only consider those sets of parameters that give rise to a phenotype similar to the profile of *Y*_*n*_ in Fig 3. We will call this profile the reference curve and denote it by *Y*_*r*_. There is nothing particularly special about this reference cure other than being more or less a typical concentration profile.

**Fig 4:**
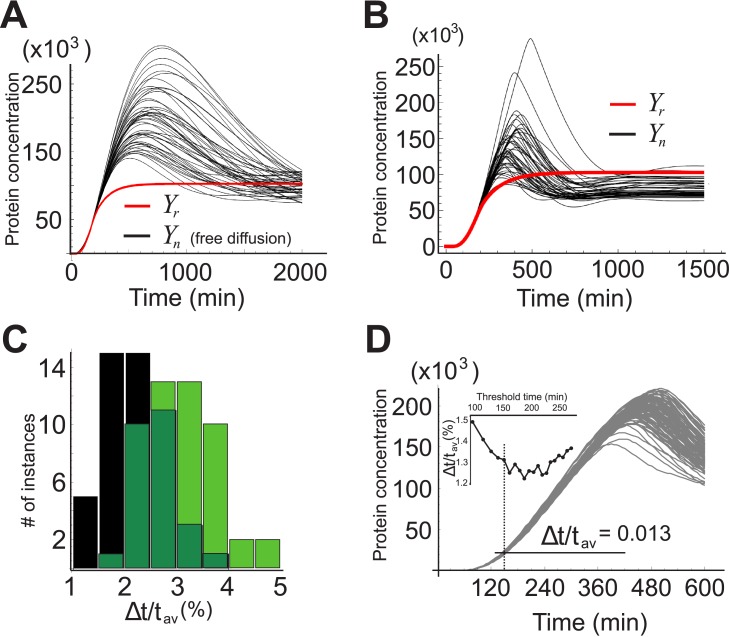
Results for the simple and the extended models. Profiles for the nuclear concentration corresponding to the 50 parameter sets for A) the simple model, and B) the extended model. In red is the reference curve *Y*_*r*_. C) Distribution of threshold times for the simple model (green) and the extended model (black). D) Superposition of 100 realizations for the best case offered by the extended model: Δ*t*/*t*_*av*_ = 0.013. The smaller plot within shows values of Δ*t*/*t*_*av*_ for different threshold times *t*_*av*_.

Since we are interested mostly in the transitional part of *Y*_*r*_, we impose that at *t* = 150 min the concentrations of all generated profiles be within 5% of *Y*_*r*_(*t* = 150 min), and that their slopes fall within 5% of ${\dot{Y}}_{r}$ (*t* = 150 min). Since we consider the steady state concentrations less important, we give them a latitude of 30%. Although we are less concerned with the profiles of all the other species, we do not want them behaving in a manner that is improbable, such as reaching unusually large steady state concentrations. To that end, we ensure that the sum of steady state concentrations of all species (proteins and their complexes) corresponding to a particular parameter set is no greater than 30% of the sum of steady state concentrations arising from the reference parameter set responsible for *Y*_*r*_. This last constraint is related to the amount of energy the cell needs to expend in order to maintain the steady state concentrations. We will discuss this point further in the “Discussion” section. The fifty profiles we obtain for the two models are shown in Fig 4A and 4B. The range of values for each parameter, along the references from which they were taken, are listed in Table 1.

**Table 1:** Range of values for all parameters.

Parameter	Range	Reference
*r*, *r*_*i*_	1–10/min	[34, 35]
*K*, *K*_*i*_	maximum: 11/min/mRNA	[34, 35]
*k*	0.5–0.005/min	[34, 35]
*q*	0.5–0.005/min	[34, 35]
*λ*	maximum 0.0534/min	[27, 36]
*q*_*i*_	0.5775–6.79 × 10^−3^/min	[37]
*γ*	0.01–0.2/min	[30]
*a*	0.01975–1.225 × 10^−4^/min	[38]
*a*_2_	0.306*a*_2_	[38]
*d*	0.015–0.102/min	[38]
*κ*	0.0248–0.426/min	[29]
*V*_*n*_(Nuclear volume)	4*μ*m^3^	[29]
*V*_*C*_(Cytoplasmic volume)	40*μ*m^3^	[29]
Number of nuclear pore complexes	80–170	[29]

Parameters (in symbols), their range, and the reference from which they were taken.

The next step is to simulate the reactions of Fig 1 using the Gillespie algorithm for each parameter set. In the standard notation, for the simple models, these reactions are:

$$\begin{array}{ccc} E+Y_{n} & \overset{w_{1}}{\to } & P_{1}\\ P_{1} & \overset{w_{-1}}{\to } & E+Y_{n}\\ P_{1}+Y_{n} & \overset{w_{1}}{\to } & P_{2}\\ P_{2} & \overset{{\tilde{w}}_{-1}}{\to } & P_{1}+Y_{n}\\ E & \overset{r}{\to } & E+X_{n}\\ X_{n} & \overset{\lambda }{\to } & X_{c}\\ X_{c} & \overset{k}{\to } & \phi \\ Y_{n} & \overset{q}{\to } & \phi \\ Y_{c} & \overset{q}{\to } & \phi \\ X_{c} & \overset{K}{\to } & X_{c}+Y_{c}\\ Y_{c} & \overset{\kappa }{\to } & Y_{n} \end{array}$$ (7)

and for the extended model they read:

$$\begin{array}{ccc} E+Y_{n} & \overset{w_{1}}{\to } & P_{1}\\ P_{1} & \overset{w_{-1}}{\to } & E+Y_{n}\\ P_{1}+Y_{n} & \overset{w_{1}}{\to } & P_{2}\\ P_{2} & \overset{{\tilde{w}}_{-1}}{\to } & P_{1}+Y_{n}\\ E & \overset{r}{\to } & E+X_{n}\\ X_{n} & \overset{\lambda }{\to } & X_{c}\\ X_{c} & \overset{k}{\to } & \phi \\ Y_{n} & \overset{q}{\to } & \phi \\ Y_{c} & \overset{q}{\to } & \phi \\ X_{c} & \overset{K}{\to } & X_{c}+Y_{c} \end{array}$$ (8)

$$\begin{array}{ccc} Y_{\alpha c}+Y_{\beta c} & \overset{a}{\to } & Z_{2}\\ Z_{2} & \overset{d}{\to } & Y_{\alpha c}+Y_{\beta c}\\ Z_{2} & \overset{q_{i}}{\to } & Y_{\alpha c}\\ Z_{2} & \overset{q_{i}}{\to } & Y_{\beta c}\\ Z_{2}+Y_{c} & \overset{a_{2}}{\to } & Z_{3}\\ Z_{3} & \overset{d}{\to } & Y_{c}+Z_{2}\\ Z_{3} & \overset{\kappa }{\to } & Y_{\alpha n}+Y_{\beta n}+Y_{n}\\ Z_{3} & \overset{q}{\to } & Z_{2}\\ Z_{3} & \overset{q_{i}}{\to } & Y_{\beta c}+Y_{c}\\ Z_{3} & \overset{q_{i}}{\to } & Y_{\alpha c}+Y_{c}\\ Y_{\alpha n} & \overset{\gamma_{1}}{\to } & Y_{\alpha c}\\ Y_{\beta n} & \overset{\gamma_{1}}{\to } & Y_{\beta c}\\ Y_{\alpha c} & \overset{\gamma_{2}}{\to } & Y_{\alpha n}\\ Y_{\beta c} & \overset{\gamma_{2}}{\to } & Y_{\beta n}\\ E_{i} & \overset{r_{i}}{\to } & E_{i}+X_{\alpha n}\\ X_{\alpha n} & \overset{\lambda }{\to } & X_{\alpha c}\\ X_{\alpha c} & \overset{k_{i}}{\to } & \phi \\ Y_{\alpha n} & \overset{q_{i}}{\to } & \phi \\ Y_{\alpha c} & \overset{q_{i}}{\to } & \phi \\ X_{\alpha c} & \overset{K_{i}}{\to } & X_{\alpha c}+Y_{\alpha c} \end{array}$$ (9)

$$\begin{array}{ccc} E_{i} & \overset{r_{i}}{\to } & E_{i}+X_{\beta n}\\ X_{\beta n} & \overset{\lambda }{\to } & X_{\beta c}\\ X_{\beta c} & \overset{k_{i}}{\to } & \phi \\ Y_{\beta n} & \overset{q_{i}}{\to } & \phi \\ Y_{\beta c} & \overset{q_{i}}{\to } & \phi \\ X_{\beta c} & \overset{K_{i}}{\to } & X_{\beta c}+Y_{\beta c} \end{array}$$ (10)

where *E* and *E*_*i*_ stand for the unoccupied promoter of the genes that code for *X*_*n*_, *X*_*αn*_ and *X*_*βn*_. From these reactions, one can obtain Eqs (1), (2), (3), (5) and (6) by using the standard assumption that the average of any product can be approximated by the product of the individual averages, e. g. ⟨*Y*_*αc*_ *Y*_*βc*_⟩ ≈ ⟨*Y*_*αc*_⟩⟨*Y*_*βc*_⟩.

What is most interesting to us is the distribution of times at which the protein concentrations of *Y*_*n*_ cross a certain threshold. The concentration threshold of 26.5 × 10^3^ is reached after the average time *t*_*av*_ = 150 min, as can be worked out from Eqs (3), (5) and (6). As with the reference curve *Y*_*r*_ *t* being 150 min is not particularly special, but such a delay does conform to what is observed in the developmental stages of eukaryotes [34, 35]. The results for all 50 sets, for both the simple and the extended models, is plotted in Fig 4C as a histogram. Appearing on the horizontal axis is the standard deviation of the threshold times Δ*t* divided by the average time *t*_*av*_ ≈ 150 min. The vertical axis shows the number of cases, out of 50, corresponding to a specific range of Δ*t*/*t*_*av*_. The superposition of histograms corresponding to the two models reveals that the extended model does better than the simple model. This may seem counterintuitive, given that the presence of importins should introduce an additional source of intrinsic noise which is not present in the simple model. Nevertheless, the degree of noise filtering achieved by both models is equally impressive. Fig 4D shows the best case scenario, belonging to the extended model, for various threshold times. There is another interesting feature to be observed here: 1) the noise during the transition, more specifically between 80 and about 300 min, is very small (see Fig 4C). 2) the noise at equilibrium is quite large, giving a Fano factor for the concentration *Y*_*n*_ of 1540. This may seem surprising: negative autoregulation is known to decrease noise. However, a limit on this effect has been shown to exist when delays are included in stochastic models [39, 40]. Although we did not add delays in our model explicitly, they are implicit in the fact that between transcription and promoter association there are several reactions that must take place. Looking at the histogram in Fig 4C, it is tempting to conclude that accuracy in timing is built into this type of system. We defer a more extensive discussion of this point for the “discussion” section.

### Delay generating circuits in prokaryotes

#### Coherent feed-forward motif

**Fig 5:**
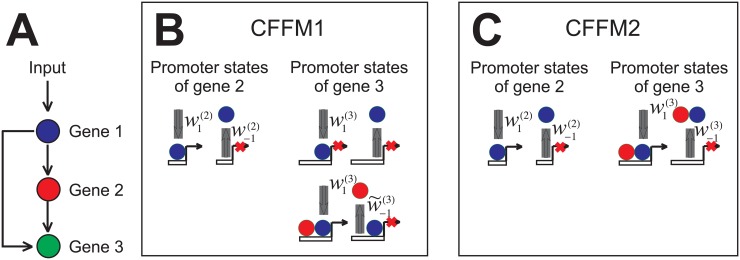
Two types of the coherent feed-forward motif. A) A schematic of the CFFM. The transcription factor of gene A (TFA) enables transcription of gene B, and TFA and TFB together enable transcription of gene C. B) First, TFA binds to the promoter of C; then, either it dissociates or TFB binds to the promoter next to TFA. TFB can bind to the promoter only when TFA is already there. Only when TFA and TFB are both in place can transcription of gene C be initiated. C) TFA and TFB form a heterodimer which then binds the promoter of gene 3, initiating its transcription.

We would like to turn attention now to gene networks which do not involve a physical barrier between different reactions, such as those found in prokaryotes, and ask the question: can these types of network mimic the temporal profile of *Y*_*r*_ of Fig 3, and if so, can they do it while maintaining such low level of noise? This question is too hypothetical to be graced with a definite answer; the number of possible motifs is too large to explore. However, we can start by looking at some delay generating motifs common to prokaryotes and see if they could be modified, e.g. by tuning their parameters, to minimize noise while keeping a temporal profile similar to *Y*_*r*_. Ideally, this (typically three-gene) motif should resemble the one of Fig 1B as closely as possible, i.e. the concentration of the protein for which gene C codes can grow only when genes A and B are on and their gene products abundant. The most common motif that fits the bill is the coherent feed-forward motif (CFFM), occurring at a frequency of 70% in *E. coli*[25]. It can come in two flavors, as depicted in Fig 5. In both cases, initiation of gene C can begin only when the transcription factors of both gene A and gene B are bound to the promoter of this gene; however, they may bind separately, one after the other (Fig 5B), or as a heterodimer (Fig 5C). In what follows, we explore both cases. For now we do not include a negative feedback; this feature will be added in the next section.

**Fig 6:**
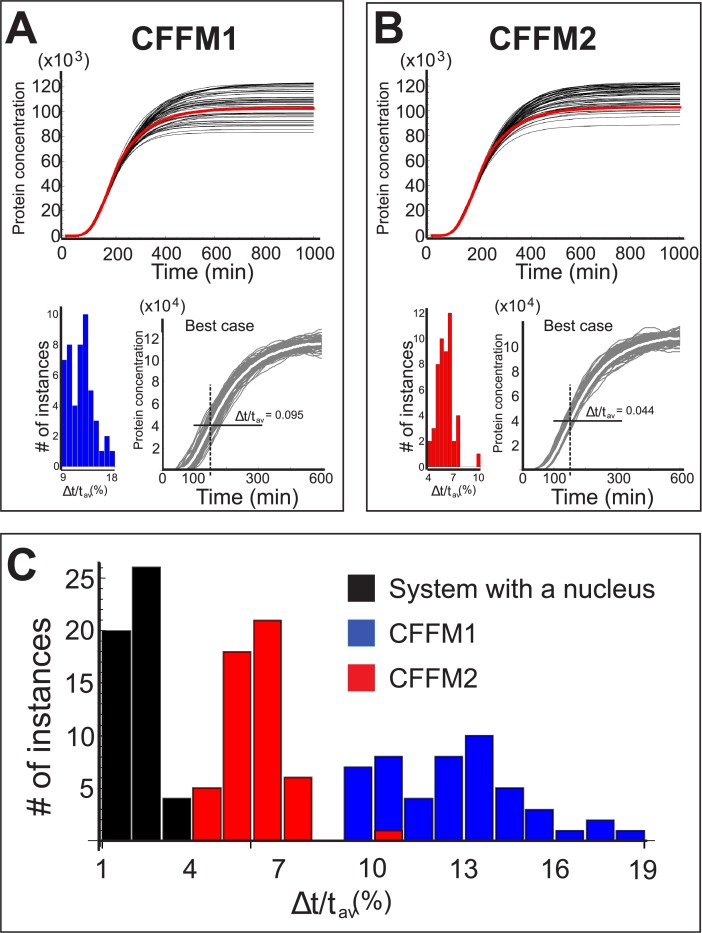
Results for CFFM1 and CFFM2. Shown here are the profiles of all fifty cases (top) and the resulting threshold times distributions (bottom), including the best case for A) CFFM1 and B) CFFM2. C) Threshold times distribution for the system with a nucleus (black) superimposed on CFFM1 (blue) and CFFM2 (red).

We are interested in making a comparison between the system shown in Fig 1B and the two versions of the CFFM of Fig 5. In particular, the temporal profiles of *Y*_*r*_ and the protein belonging to gene C should be close. To make this comparison as fair as possible, we apply the same conditions and constraints as in the previous section. The reader should refer to this section and merely replace Eqs (3), (5) and (6) with the following equations:

$$\begin{array}{ccc} {\dot{X}}_{1} & = & r_{1}-k_{1}X_{1}\\ {\dot{X}}_{2} & = & r_{2}S-k_{2}X_{2}\\ {\dot{X}}_{3} & = & r_{3}P_{2}-k_{3}X_{3}\\ {\dot{Y}}_{1} & = & K_{1}X_{1}-q_{1}Y_{1}\\ {\dot{Y}}_{2} & = & K_{2}X_{2}-q_{2}Y_{2}\\ {\dot{Y}}_{3} & = & K_{3}X_{3}-q_{3}Y_{3}\\ \dot{S} & = & w_{1}^{\left(2\right)}Y_{1}\left(1-S\right)-w_{-1}^{\left(2\right)}S\\ {\dot{P}}_{1} & = & w_{1}^{\left(3\right)}Y_{1}\left(1-P_{1}-P_{2}\right)+{\tilde{w}}_{-1}^{\left(3\right)}P_{2}-w_{1}^{\left(3\right)}Y_{2}P_{1}-w_{-1}^{\left(3\right)}P_{1}\\ {\dot{P}}_{2} & = & w_{1}^{\left(3\right)}Y_{2}P_{1}-{\tilde{w}}_{-1}^{\left(3\right)}P_{2} \end{array}$$ (11)

for CFFM1, and

$$\begin{array}{ccc} {\dot{X}}_{1} & = & r-k_{1}X_{1}\\ {\dot{X}}_{2} & = & rS-k_{1}X_{2}\\ {\dot{X}}_{3} & = & r_{3}P-k_{3}X_{3}\\ {\dot{Y}}_{1} & = & KX_{1}-qY_{1}\\ {\dot{Y}}_{2} & = & KX_{2}-qY_{2}\\ {\dot{Y}}_{3} & = & K_{3}X_{3}-q_{3}Y_{3}\\ \dot{Z} & = & aY_{1}Y_{2}+w_{-1}^{\left(3\right)}P-w_{1}^{\left(3\right)}Z\left(1-P\right)-\left(b+2q\right)Z\\ \dot{S} & = & w_{1}^{\left(2\right)}Y_{1}\left(1-S\right)-w_{-1}^{\left(2\right)}S\\ \dot{P} & = & w_{1}^{\left(3\right)}Z\left(1-P\right)-w_{-1}^{\left(3\right)}P \end{array}$$ (12)

for CFFM2. As before, the variables *X* and *Y* denote mRNA and protein concentrations respectively, with the subscript indicating the gene, and *Z* refers to the heterodimer comprising of *Y*_1_ and *Y*_2_. For CFFM1, the association and dissociation rates between the protein (either monomer or heterodimer) and a gene’s promoter are given by $w_{1}^{\left(i\right)}$ and $w_{-1}^{\left(i\right)}$ respectively, with the upper index indicating the gene: *i* = A,B,C. The rate at which a protein encoded by gene A binds with that of gene B is represented by *a*, while the rate of their dissociation is given by *b*. In deriving Eqs (11) and (12), we made the same assumptions (those relevant to the present system) listed at the beginning of section “Deterministic model”. When all the steps of the previous section are carried out, the results are as presented in Fig 6.

The difference between the system of Fig 1B and the two types of the CFFM is immediately apparent: the distribution of Δ*t*/*t*_*av*_ for CFFM1 and CFFM2 is shifted to the right; the first is also more spread out. Interestingly, the best case for CFFM2 is nearly as good as the worst case for the system with a nucleus and importins.

#### Variations on the coherent feed-forward motif

Since in the nucleus bearing system the gene of interest auto-repressed itself, it seems only fair that we add such a feature to the CFFM. Instead of considering only the case where gene C represses itself, we look at two other variations on negative feedback within the CFFM: gene C represses gene A; and gene C represses gene B. The equations governing the promoter occupancy of the gene being repressed are the following:

Gene C represses gene A (CFFM1(-)):
  $$\begin{array}{ccc} {\dot{P}}_{1} & = & w_{1}^{\left(1\right)}Y_{3}\left(1-P_{1}-P_{2}\right)+{\tilde{w}}_{-1}^{\left(1\right)}P_{2}-w_{1}^{\left(1\right)}Y_{3}P_{1}-w_{-1}^{\left(1\right)}P_{1}\\ {\dot{w}}_{2} & = & w_{1}^{\left(1\right)}Y_{3}P_{1}-{\tilde{w}}_{-1}^{\left(1\right)}P_{2} \end{array}$$ (13)
Gene C represses gene B (CFFM2(-)):
  $$\begin{array}{ccc} \dot{S} & = & w_{1}^{\left(2\right)}Y_{1}\left(1-S-P_{1}-P_{2}\right)-w_{-1}^{\left(2\right)}p_{1}\\ {\dot{P}}_{1} & = & w_{1}^{\left(2\right)}Y_{3}\left(1-S-P_{1}-P_{2}\right)+{\tilde{w}}_{-1}^{\left(2\right)}P_{2}-w_{1}^{\left(2\right)}Y_{3}P_{1}-w_{1}^{\left(2\right)}P_{1}\\ {\dot{P}}_{2} & = & w_{1}^{\left(2\right)}Y_{3}P_{1}-{\tilde{w}}_{-1}^{\left(2\right)}P_{2} \end{array}$$ (14)
Gene C represses itself (CFFM3(-)):
  $$\begin{array}{ccc} {\dot{P}}_{1} & = & w_{1}^{\left(3\right)}Y_{1}\left(1-P_{1}-P_{2}-P_{3}-P_{4}\right)+w_{-1}^{\left(3\right)}P_{2}-w_{1}^{\left(3\right)}Y_{2}P_{1}-w_{-1}^{\left(3\right)}P_{1}\\ {\dot{P}}_{2} & = & w_{1}^{\left(3\right)}Y_{2}P_{1}-{\tilde{w}}_{-1}P_{2}\\ {\dot{P}}_{3} & = & w_{1}^{\left(3\right)}Y_{3}\left(1-P_{1}-P_{2}-P_{3}-P_{4}\right)+{\tilde{w}}_{1}^{\left(3\right)}P_{4}-w_{1}^{\left(3\right)}Y_{3}P_{3}-w_{-1}^{\left(3\right)}P_{3}\\ {\dot{P}}_{4} & = & w_{1}^{\left(3\right)}Y_{3}w_{1}-{\tilde{w}}_{-1}^{\left(3\right)}P_{4} \end{array}$$ (15)

**Fig 7:**
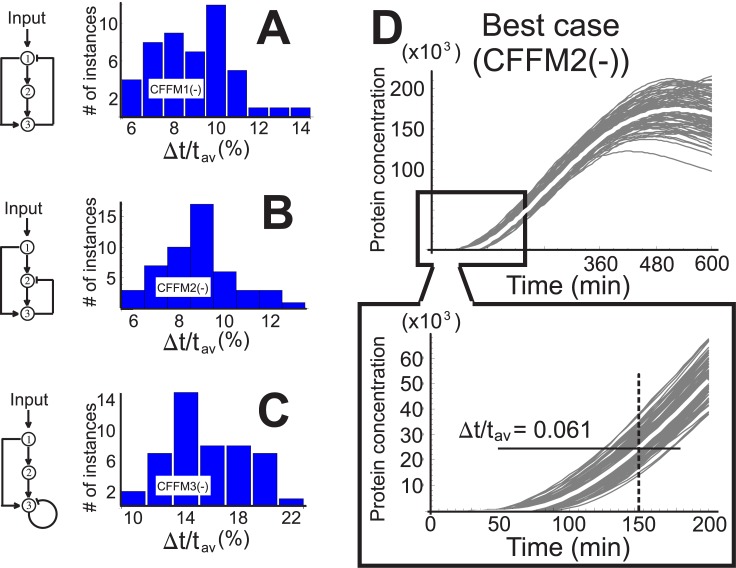
Three variations on the coherent feed-forward motif. The histograms in A, B and C show the distributions for the three variations on the CFFM the schematics of which are to the left. In all three cases, the transcription factors bind to the different promoters one at a time: no (hetero) dimers. D) Best case, corresponding to CFFM2(-) with Δ*t*/*t*_*av*_ = 0.061.

The new variables *P*_1_ and *P*_2_ refer to the promoter occupancy of gene C by one and two copies of *Y*_3_ respectively. Repeating the now familiar procedures of sections “Stochastic model” and “Coherent feed-forward motif”, renders the results shown in Fig 7. The profile for the best case, corresponding to the motif CFFM2(-), is shown in Fig 7D. The quantity Δ*t*/*t*_*av*_ in this case is 2 times greater than what it is for the worst case for the system with a nucleus, which is Δ*t*/*t*_*av*_ = 0.05, and 6 times greater than its best case (Fig 4B). The eukaryotic motif wins out again.

## Discussion

We have shown that in the eukaryotic cell, a negative feedback combined with an import of proteins from cytoplasm to nucleus, either active or diffusive, can not only lead to a delayed switch-like transition, but also to a highly precise timing of this transition. Conventionally, a negative feedback has been used to speed up the transition into a steady state, not slow it down. However, the presence of a nuclear barrier ensures that proteins inside the nucleus do not amass until the proteins in the cytoplasm gain abundance. Once they do, the nucleic proteins start growing at a large rate, giving rise to a switch-like behavior. As for the timing accuracy, this is likely a result of the fact that when a process, e.g. protein synthesis, consists of many steps, the uncertainty in “when” does the last step occur is inversely proportional to the number of steps [41]. In the present case, going from mRNA to nucleic protein, takes four steps, for the simple model, and five steps for the extended model (refer to Fig 1).

**Fig 8:**
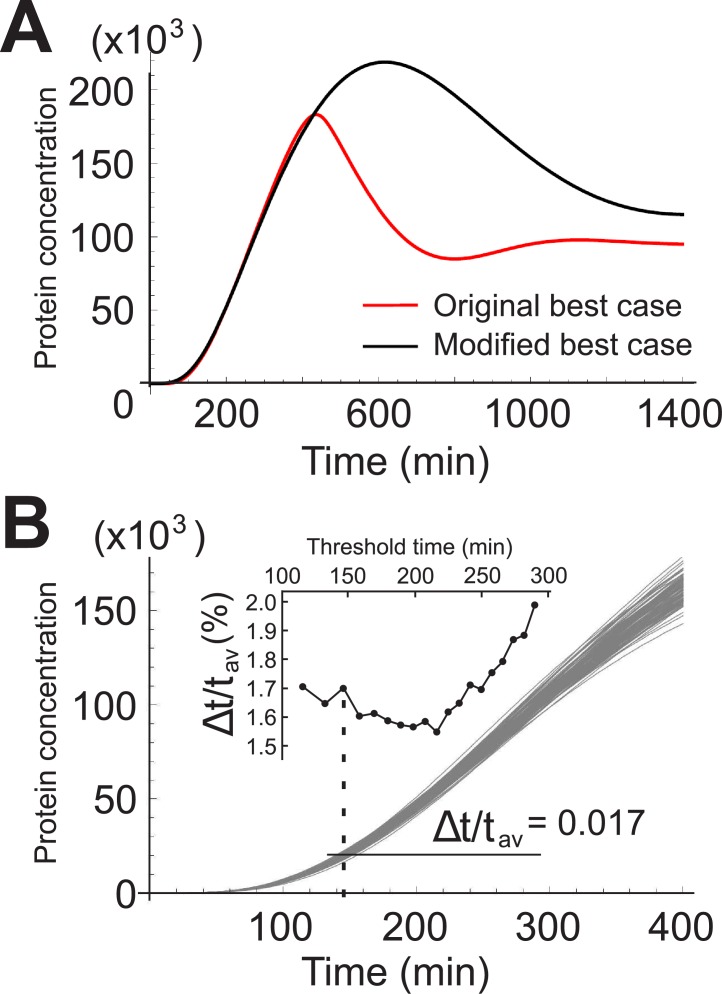
Importins in a steady state at *t* = 0. A) Average protein concentrations for *Y*_*n*_. The red curve corresponds to the best case discussed in Fig 4. The black curve is the profile obtained when the concentrations *X*_*αn*_, *X*_*αc*_, *X*_*βn*_, *X*_*βc*_, *Y*_*αn*_, *Y*_*αc*_, *Y*_*βn*_, *Y*_*βc*_ and *Z*_2_ start out in a steady state, and *q* → *q*/2.4 and *a*_2_ → *a*_2_/50. B) 100 profiles generated by the Gillespie algorithm. At *t* = 150, Δ*t*/*t*_*av*_ = 0.017.

We note that the results of the extended model rely on the requirement that all three genes are turned on at the same time. In real systems this may not always be the case. In many situations the importins may have reached steady state well before the gene of interest is turned on. In fact, experimental studies reveal that neither case is strictly true; the importins themselves are regulated by other molecules, making their concentration vary over time [31–33]. For the sake of completion, however, we did consider a situation where, at time *t* = 0, all the gene products pertaining to importins are in a steady state. As an example, we took the parameters corresponding to the best case scenario, shown in Fig 4D, and modified them such that the temporal profile of *Y*_*n*_ under the new conditions matches the reference profile *Y*_*r*_ according to the constraints of section “Stochastic model”. To have a good match, we only had to let *q* → *q*/2.4 and *a*_2_ → *a*_2_/50. Fig 8 shows the result. The temporal precision, while still very good, is diminished by about 30%. This result does not necessarily mean that the precision is always diminished for the latter, as the parameters for the best case scenario may be completely different. An analysis similar to the one carried out in section “Stochastic model” will be necessary in order to determine whether starting out with importins, and the mRNAs from which they are translated, already in a steady state follow the distribution similar to the ones in Fig 4C. We leave this analysis for the future. The proven capability to control importins experimentally [42] makes our results testable.

With regards to the prokaryotic cell, we asked whether a genetic motif could be constructed such that its protein output would mimic the output of the nucleic protein. We considered two types of the coherent feed-forward motif, and three variations on it, and showed that while all of these motifs can be tuned to reproduce the temporal profile of the nucleic protein concentration, they cannot compete with the eukaryotic motif when it comes to temporal precision. The difference is not merely noticeable; it is remarkable. In the best case scenario, provided by the motif CFFM2, the relative deviation from the average threshold time amounted to 4.4%, a value 3.5 times greater than that found in the eukaryotic cell.

These findings suggest that one of the functions of the cell nucleus is to regulate fluctuations in protein abundance during transitions into steady states. From our limited number of cases, it appears that cells without a nucleus (and importins) do not have the means to reduce these fluctuations enough in order to effectuate reliable changes in any genetic program downstream. There may be, of course, larger motifs consisting of many genes that may cater to one specific gene in order to reduce noise associated with its corresponding protein. However, such gene regulatory networks, while conceivable, would demand a lot of energy to function. Research suggests [43] that early in the history of eukaryotes, energy was an important selection factor; networks that required less energy were more likely to persist. This begs the question: could organisms with complex spatio-temporal patterns have evolved without a nucleus? Or is the fact that in billions of years prokaryotes have not evolved into such organisms just a coincidence?

## Supporting Information

S1 File Lists of parameters for all the models and cases. The parameters are ordered as indicated in the “Table”. Where applicable, other information is provided under each “Table”.
(DOC)
