# Supplementary material for: Is the Cell Nucleus a Necessary Component in Precise Temporal Patterning?
Source: PLoS One. 2015 Jul 30;10(7):e0134239. doi: 10.1371/journal.pone.0134239 (PMC4520485; doi:10.1371/journal.pone.0134239)
Supplement: S1 File — The parameters are ordered as indicated in the “Table”. Where applicable, other information is provided under each “Table”. (DOC) [file pone.0134239.s001.doc]

Supplementary Material

**In the tables below we list all the parameters used to generate both deterministic and stochastic results. The parameters are ordered as indicated in the table labeled “Table”. Where applicable, other information is provided under each “Table”.**

System with a nucleus + improtins

**Table={r_i, K_i, k_i, q_i, a, d, \lambda, \gamma_1, w_1, \kappa, r, K, k, q}**

**w_{-1}=1; \tilde{w}_{-1}=1/10, a2=0.306 a.**

**{{3.7011917074490537`,0.7561459474976271`,0.017349388764987442`,0.003588364114301602`,0.00006315565702850727`,0.06894930125952287`,0.03104803441428676`,0.19934814002104584`,6.62456950152001`*^-6,0.3828583466919506`,7.449944926081912`,4.519106938090868`,0.005942583743465665`,0.006305248373885561`},{4.362210865523991`,1.430254955699339`,0.030700919505887464`,0.004702241860606697`,0.0001585629661954905`,0.008628016355106516`,0.03103602825344713`,0.08329527432421435`,5.616760001932936`*^-6,0.22135693749352725`,6.45371183501079`,1.5700962895993475`,0.006180438930246335`,0.006648132566223305`},{3.188976320652179`,2.1741955029950404`,0.02719721965017946`,0.006867573548978577`,0.00010626309977155161`,0.06649381360021711`,0.019379168885984996`,0.18633186859012835`,6.386757598326673`*^-6,0.13728614874529332`,8.872463707691466`,4.270153534743438`,0.007084970911378899`,0.011089662204767523`},{6.001643841608203`,1.7441954028396554`,0.05644034194405659`,0.004652671009789863`,0.000027504794359699492`,0.016189288374209743`,0.027805424575999882`,0.10776260338516597`,8.10747549488484`*^-6,0.38172332707691103`,9.637834615389401`,4.056244210079315`,0.011754505826023526`,0.005728928620659032`},{8.537445721594707`,1.3344695161885252`,0.042313945440318135`,0.004965546945949408`,0.000060563206895316305`,0.10323805330113522`,0.023752352938807605`,0.044747347094266116`,9.827470013353681`*^-6,0.17867403722251363`,7.588114844447498`,4.65930406237201`,0.006391826624807487`,0.005710053007545499`},{6.152202935963478`,1.647066741682825`,0.03858261185733647`,0.004460309736340173`,0.00013084037444497528`,0.17945948265438572`,0.010942522135563043`,0.14192569919714026`,8.11831107610558`*^-6,0.4275225669645144`,8.416242692147971`,4.696761650984229`,0.005043847077063179`,0.013425112487708293`},{3.0567791745758264`,3.5491209333040725`,0.05569172991430335`,0.005341396904590997`,0.0000837114834533434`,0.07945559751223802`,0.020451169705128724`,0.17763613853419424`,5.685083181862154`*^-6,0.14401048093774316`,8.333119413588719`,4.9339950390528475`,0.007302558785528657`,0.007214370320233254`},{7.195212478080576`,1.8429428225442868`,0.09410256868048247`,0.005736580201469881`,0.000026149315037068152`,0.1411013179504404`,0.041426440693181965`,0.15602096595696335`,6.1716729943627205`*^-6,0.4735031556232381`,9.368530481559606`,4.829165431394459`,0.011560911328494142`,0.005291539338483794`},{5.809263288240631`,3.5327382999356978`,0.08153560377274596`,0.004436788945558499`,0.0001990054804953565`,0.043061418280446434`,0.010141865987014012`,0.13588998489365628`,6.100428468359584`*^-6,0.21126358287174474`,9.545037920080325`,2.8666682361606455`,0.005766114630573138`,0.012807289613160244`},{3.9228008354171333`,2.589697175458017`,0.05037968129313122`,0.004816582650911992`,0.00015038045114206517`,0.14927988752333915`,0.017905817921863854`,0.18842903410906509`,7.0994836488301155`*^-6,0.1364574768737511`,8.741791229229555`,3.683231922927609`,0.005786830475424498`,0.007960811004379481`},{4.987303992679191`,1.540260985576447`,0.04417410200198865`,0.0048014208713334375`,0.00008631505314685549`,0.18415085789161462`,0.023584707629681442`,0.09724370353436507`,6.845749383879388`*^-6,0.39058770025672684`,8.288262112339957`,4.645249461969868`,0.00541363946050237`,0.00847080645797936`},{3.0501492373385553`,1.1452391800882245`,0.009772552866211412`,0.006271115879363322`,0.00008968606751839629`,0.04963549386223157`,0.0323389200145386`,0.1589088441532847`,5.685464467723056`*^-6,0.15541164890832126`,5.264317815258416`,3.4796359333269944`,0.009669348031749875`,0.005496853160801396`},{5.93099414350376`,1.8409261773520713`,0.05399867835174807`,0.0058386934434009235`,0.00010947644497678885`,0.09430259450668133`,0.01301504153856288`,0.1600618857206394`,8.912306846274428`*^-6,0.19581856643774953`,8.882341218520288`,4.983115334867934`,0.012727793403585971`,0.005203782296199305`},{2.403509770184524`,2.927285440485126`,0.04911958056040483`,0.003502458449413594`,0.0000855519513068801`,0.1910740224983581`,0.01742723978022931`,0.17681988881033467`,5.982033203490082`*^-6,0.3792631418669673`,6.003899164877989`,4.635441531010185`,0.0073121160926407636`,0.0065543130250376586`},{2.1192876089027344`,3.6286382391845535`,0.07763211803729356`,0.006145534675421809`,0.00016769275099390055`,0.13789943519657966`,0.026481163427397078`,0.1782258291432139`,5.0724634802423164`*^-6,0.47149627500277924`,5.938485120451283`,2.8816122808111677`,0.007642888926184782`,0.0059797016714398105`},{5.990781000995408`,1.5069077852481287`,0.08123522893367349`,0.0018155656453419438`,0.00003171646854811805`,0.015405802996166673`,0.04508262422148107`,0.18154211785341945`,6.227341252316168`*^-6,0.4134990735563213`,8.436270165357431`,2.9020136733071187`,0.013048744959789332`,0.006392594339931265`},{5.44344145055228`,2.5348934702669474`,0.08959564636623077`,0.0032871988709726454`,0.000087838429012715`,0.08084364930238014`,0.018444769624516938`,0.09349550076043295`,7.806161848738089`*^-6,0.33715000852425014`,9.119088562262192`,3.5034540234289864`,0.007392906386507317`,0.007664190964796508`},{2.9602749023772574`,2.730928227687145`,0.04244397982000164`,0.004521299740507286`,0.00004750740829763658`,0.028917918841182222`,0.026402135549034145`,0.19165101058452888`,9.30481038417614`*^-6,0.14220589168168873`,9.512900841006573`,3.9250457139150363`,0.006643467573061501`,0.006750196452654372`},{1.7912390626721066`,2.9125632255932743`,0.035242831332319785`,0.0028485152416643485`,0.000027993616101604468`,0.029462798401050566`,0.042714923186891976`,0.1845025839916793`,6.163888422532986`*^-6,0.4580252087856702`,8.296149493287231`,4.7448495738336955`,0.011107819376555764`,0.012051476649419024`},{6.207052990629739`,3.4282239789432065`,0.09091091055253175`,0.003880974186022128`,0.0001205308005693297`,0.1312026487689189`,0.009949248493295773`,0.0962959779786875`,5.477321934864185`*^-6,0.37565023587520885`,9.490988671993176`,3.8201971230917113`,0.02462936585928087`,0.005004062393371547`},{2.370803895759588`,3.8941133745634833`,0.08666630425921149`,0.0035294228264486334`,0.00005598935352834627`,0.09310035359551933`,0.05819295105675787`,0.14996839768262998`,7.105945848838775`*^-6,0.48012614459185854`,8.009894866504617`,1.3635105860779753`,0.005115922138506979`,0.005193449323905239`},{2.451477729606026`,1.6755142817605115`,0.019065211142189703`,0.005181765684461308`,0.00006756385110370173`,0.08734893583752151`,0.03255888456870479`,0.1483860260584588`,6.0560195971852655`*^-6,0.24622793251245856`,8.24660244508761`,3.3313854758195216`,0.008536040670904368`,0.007770758751605961`},{6.841953993974384`,3.1958267823668773`,0.09735988013779026`,0.004867730235625595`,0.00010282287353175691`,0.04036551500934024`,0.011993406882569002`,0.13523321907454217`,7.668934620076739`*^-6,0.17024030531242795`,7.927688923166764`,4.7933834343690815`,0.0070949615760987905`,0.007519833238346471`},{5.150654771254599`,1.4148546361770906`,0.07884215952826143`,0.001878037256399275`,0.00017168421315892784`,0.024008517516849653`,0.03328629306608896`,0.18353389409195625`,5.759261330453842`*^-6,0.3148905870497556`,5.962460721226291`,3.859840632335837`,0.006709201957765559`,0.01015608329936718`},{5.354343530604957`,1.8106968742340195`,0.04153399901005286`,0.004319961933435648`,0.0001393860065675545`,0.18565708331036312`,0.01615435495561121`,0.17302837081635297`,5.089087054794259`*^-6,0.2695275445038221`,6.6273959303686105`,3.033801416149667`,0.010604653948189283`,0.005934198574497854`},{2.533580818082118`,4.699333085971194`,0.09768514655368374`,0.0010533081530780947`,0.00006283328042850373`,0.14218178301591378`,0.022286283155162984`,0.13489770792663386`,6.234742897050024`*^-6,0.4840095580222811`,6.4144061918352815`,4.39519934965344`,0.011464712114353043`,0.006407126119100187`},{5.329210975798514`,3.8365982724476826`,0.06547789760073178`,0.006579045805632158`,0.00010409411667651011`,0.10028910021049053`,0.0122136876006219`,0.05552861716632218`,0.00003751282341463785`,0.16405759185893454`,7.629762045483638`,3.753305698975204`,0.005024656252515927`,0.005141864659872694`},{2.4597557394164937`,1.339634636521045`,0.02070250090997877`,0.006792420748329329`,0.00015061773186783006`,0.05389996666686275`,0.03802300105052672`,0.14160155673607383`,5.791053468652404`*^-6,0.36097261237342215`,6.727485269785602`,4.335436447012249`,0.011202979636135604`,0.007630278248566588`},{6.484847400964224`,2.184980863487131`,0.09937688566105315`,0.0026816914913116103`,0.000025832498192075314`,0.0509562301949644`,0.052051165532091825`,0.08501917844913981`,8.680053986735489`*^-6,0.4715994528336882`,4.732193891969494`,4.8329693748052485`,0.0069848898310130025`,0.005734464338036857`},{3.5457350861791372`,1.665533149341111`,0.08166626775738323`,0.0013611759002671083`,0.0001558972824528278`,0.015270880760493655`,0.05879017326923132`,0.13745006860882636`,7.373674175208904`*^-6,0.43960810762717184`,8.472419255297119`,3.344233095725812`,0.011729744103311276`,0.00622572701591706`},{1.8694481064359767`,3.1680427366364246`,0.0266891173631697`,0.0057564192141822115`,0.00011571783663084275`,0.15353031278477258`,0.02046551312752279`,0.09100177911946117`,5.9826933678013996`*^-6,0.4013149935082315`,8.536412605857073`,2.5742451416601213`,0.012515870091038217`,0.005308134977399598`},{2.2798111388714624`,1.2927996266532116`,0.01770437128600086`,0.005514594547914818`,0.00010097980912295791`,0.18479817796190456`,0.053607911895151614`,0.16505059213939643`,7.273651649228232`*^-6,0.2538956036435508`,9.126378496589954`,3.928687597273038`,0.006555300031570524`,0.009105470447583267`},{1.8928383274288976`,3.3084815081691588`,0.05379441937188473`,0.002699248736340659`,0.00008568586651515263`,0.019878127085928565`,0.04613803295822`,0.167796013084049`,5.630724175632189`*^-6,0.18747671996377568`,3.6852616177822526`,4.510474650355825`,0.0114091372493752`,0.005269398617835527`},{1.9562183201936776`,2.0185004009776346`,0.03222928319886703`,0.0037220021671924815`,0.00014569322555345627`,0.14422495815704656`,0.04013333357664252`,0.18229122154504063`,6.005515135775054`*^-6,0.27020954940210584`,9.445174970808928`,4.021235542671564`,0.005052959462045731`,0.010002162319101615`},{9.248952796918012`,1.604875204951819`,0.067292980366635`,0.005884855063308248`,0.00003354210532316353`,0.03072431173543208`,0.01598267723619258`,0.08366854178898275`,6.084159637243713`*^-6,0.47389733501048403`,8.357302304336297`,4.669969413939498`,0.011131695008562134`,0.006031781704178285`},{6.695339485682355`,2.73762178060427`,0.053137600906800114`,0.005998682230735669`,0.0001396964067854012`,0.18787430065672855`,0.007951090947868833`,0.08160548938870293`,9.16202391587648`*^-6,0.4283144579287649`,7.468701072225315`,3.8214916078231145`,0.005490616677961746`,0.005665941774624511`},{3.9528229498740224`,4.499092691786334`,0.052734651095361296`,0.006054880148018229`,0.00004310496661784436`,0.013877737657254252`,0.01548063817282691`,0.09203524034825816`,6.146782416616832`*^-6,0.18166696304772745`,8.574987245501621`,2.8551810438036593`,0.0071564968542970064`,0.010536050943833405`},{3.659779329938651`,4.16499289905906`,0.06470748614913255`,0.005324365792739116`,0.00007282883449901114`,0.02607087735338584`,0.012760782282435781`,0.14580242621697792`,5.078632005720972`*^-6,0.25798666305478013`,9.022298519560064`,3.800098886517759`,0.005993981541442317`,0.01410331456740839`},{2.336566653219382`,4.258936911573258`,0.06223125385789824`,0.0034428848975356008`,0.00002541855136092532`,0.05753882105148278`,0.02527356587247287`,0.16878363881385877`,5.670584679181697`*^-6,0.3803742880214891`,7.125418883994751`,4.253223066681748`,0.01182671610813485`,0.005581687935956078`},{2.719829757543838`,2.7431594907111707`,0.08941238282700831`,0.002921470728437751`,0.0001231284386431654`,0.19023012130621664`,0.0513772790935564`,0.14137584132937758`,7.29171756308431`*^-6,0.42230130963191326`,7.1865248019791395`,4.662136554189193`,0.010603816921536419`,0.005460788721678711`},{4.239544290705762`,1.2778898490683268`,0.022120866180145055`,0.005893173252284652`,0.00005584797440180147`,0.07489527600957696`,0.03897132327495681`,0.14684591086473586`,7.163410885240233`*^-6,0.1177255161121219`,6.64248328058334`,3.9820979269287733`,0.006687648897697213`,0.0061053417868431326`},{4.679998216272578`,2.4178491677194422`,0.09075310113309912`,0.002918072940184125`,0.00006800961749666812`,0.03856260737794698`,0.02033365651451978`,0.18061416947633224`,7.990782897028739`*^-6,0.44425511542391016`,6.9550796615752635`,2.6694714734551974`,0.00843741554296494`,0.005077241296531226`},{1.4581150394126077`,2.5291402764834716`,0.02511706522522636`,0.006050481774325984`,0.00014538974403544548`,0.18488446772869116`,0.04073689617711787`,0.0906465324714803`,5.05647952325256`*^-6,0.4006404335959367`,9.507674055923331`,4.659359765920378`,0.013437241300005134`,0.005155035837566269`},{7.911000603155355`,1.6406820809899276`,0.04761868158161542`,0.004848389598170934`,0.0001410596319583133`,0.15459641366860155`,0.011104630819885539`,0.06906517824189135`,6.811942573854606`*^-6,0.44853916228013135`,8.812699308051215`,2.8631734660223893`,0.00724741476877816`,0.005208954141055544`},{3.238025949680498`,1.2828803014920664`,0.03963961181095946`,0.004081132142191143`,0.00016208888245630235`,0.0891400362399914`,0.040686167075483254`,0.12505600822188484`,5.28750527050626`*^-6,0.371378132279769`,8.802342796456688`,4.896480725476486`,0.01737910011088005`,0.006323948058894552`},{4.48532341299957`,0.8049971558795921`,0.03675612979770995`,0.00205002025205392`,0.00010294535195868516`,0.02698490330577527`,0.03935948316083296`,0.16984867228691497`,8.128770698603903`*^-6,0.30495748177964166`,9.16004367968025`,4.0795181785461825`,0.005263884587006121`,0.005349632878208055`},{1.9031075573345948`,3.2845234510868817`,0.04786708806865486`,0.0056851646027638045`,0.00009789663709751088`,0.09668454481975836`,0.03127165626550403`,0.14053275385834063`,5.276996389845834`*^-6,0.3577554541478285`,5.271409530615204`,3.091946478128513`,0.006075915740686694`,0.0055263219703334115`},{6.98543417302775`,0.458001353020717`,0.019537292550407356`,0.006878681212713555`,0.00008527983573406706`,0.05648352511885363`,0.05603933961855734`,0.1964978309893644`,5.688365299700692`*^-6,0.20290974774406134`,8.89477328918085`,4.552286270581748`,0.009161694173759574`,0.0070277901948291865`},{7.98611587527599`,1.1253881471977838`,0.03138829201178049`,0.00684384375182199`,0.000037503725360536836`,0.037193281751209395`,0.019340531910868708`,0.1122412716876291`,5.269266670061528`*^-6,0.4888397828444746`,4.321253368520061`,3.9310176617478607`,0.00731552466302568`,0.00865124337433068`},{8.508510294335707`,1.8209222282713968`,0.09515967009521459`,0.0053263121141210795`,0.00009335778542275737`,0.03975555122431415`,0.018608773170954676`,0.14256904189644581`,0.000011472860948210159`,0.24198001837975924`,8.321957535897688`,4.861294529504001`,0.008002577123848512`,0.008298542208972456`}};**

System with a nucleus - no improtins

**Table={r, w_1, \lambda, k,\kappa, K, q}**

**w_{-1}=1, \tilde{w}_{-1}=1/10.**

**{{4.40931332522056,0.000022318132570480335,0.008130955405245333,0.002229828316290101,0.12804954020024337,2.609918481271479,0.0012857785319296156},{2.5622420968600594,0.000009737503920847852,0.009127958423083405,0.003735453569194185,0.13994351366564614,4.057575326273103,0.0018945344647582284},{3.7545725656350437,0.000006620298998965595,0.010171894007083538,0.003655006899993474,0.17543095325706723,2.596036403895801,0.0022668989686674764},{10.292768841661298,0.000021581017073680047,0.006818379535751618,0.0020435319975095913,0.18691515645431186,1.2709298640290978,0.0017766570121202827},{9.975119718254861,0.000013704109300370632,0.010508490618351997,0.0027525830741161974,0.1258765112088463,0.9793450264830739,0.0012187415359191758},{2.1663232075401875,0.000007364668433829521,0.011443913586578258,0.0033035092943647995,0.11568597154975979,4.228538591871287,0.0017936869277208772},{7.858524585827613,0.000012826606786398936,0.005671151579926814,0.01116033764012654,0.1726396641267349,2.525690354095069,0.0008350546963771324},{4.493857667794067,0.00000768026684990133,0.005439319290603351,0.00859966405589618,0.2845411939893577,4.082571402504717,0.0017400574879174676},{6.768361359229787,0.000013665871156064579,0.006275082166520951,0.0075000954620400954,0.13134696025469003,2.3797875452639694,0.0007848342610747614},{4.821346056949187,0.000011522506062420277,0.011601650240136686,0.005836673783965223,0.13985887907071387,2.1167750614223424,0.0015437129643832656},{3.4580720097041264,0.000010202892696328139,0.014590797893360109,0.002608178730894881,0.13302362729513892,2.3806956198485976,0.0022863551376415465},{3.704989624687716,0.000009369844190652349,0.007038178589297939,0.007560627475719528,0.23242202819338165,3.960544671865941,0.0015592975943886037},{5.684149864772019,0.000008892781305260815,0.005790676171665954,0.0058054181405633185,0.23747794988603932,2.6804178150245246,0.0017695538847528347},{6.7846363732931,0.000011560519595550379,0.00921389864162574,0.007006978414393803,0.0933071870369028,1.893941092884825,0.0010346087879346659},{3.432272804544656,0.00001543033624532311,0.013530844458481584,0.005067158535487133,0.09766544637873459,2.7632189432326895,0.001102558578901673},{8.938147574621324,0.000013660724839986279,0.006059799333171423,0.003254833494168574,0.2751968755638742,1.625817448737048,0.0023407562088469673},{6.239255645302729,0.000011293852550850502,0.005217779018531221,0.004949574533474728,0.20854483343791436,2.6314924179037913,0.0013924377584184722},{4.956401356495034,0.00001480972230934361,0.005483784775666578,0.003317795972024742,0.12276876680109297,3.273906767873246,0.002261496509536685},{3.2528444718531846,0.000020197220774495402,0.005098166973994101,0.002197615261050716,0.34002430151098456,4.989450940536104,0.0022258819829436955},{7.253590144896904,0.000009782645867751683,0.00629402591604662,0.009091672176393492,0.1757329822641463,2.3573869797504594,0.0016853726120076927},{6.0813174830264485,0.000006176971442264892,0.013209855212170841,0.005203609137291592,0.09329761655300627,1.5938129309151998,0.0024320271205745384},{9.706412240824246,0.000034371400716731024,0.007100597651373697,0.006632098337111509,0.08424563903901167,1.6623599551973562,0.00051974876089696},{4.0586333982546074,0.00001429789160751036,0.005649340995038799,0.005970230504660448,0.08262150891537293,4.3815126794364225,0.0010564534467454468},{2.7633470909439115,0.000014717271907489105,0.009297796186461848,0.004603665073573291,0.11284702532285294,4.1151627848734975,0.0012228546738635494},{6.360221315360646,0.000013580627833395368,0.006337772173073431,0.005986916872132788,0.1491472989856277,2.3695944429684017,0.0013396941660736355},{5.508855863373859,0.000008466869003352891,0.007141910625308646,0.011385270691748063,0.09222614638414157,3.3498087500971527,0.002155203457540411},{2.3229300783778792,0.000011395370127937383,0.010877882294765096,0.004511350666021085,0.369336332727505,4.057847049538291,0.001334051489416174},{2.2511566136383445,0.000007329402345192665,0.009963770572716425,0.004377721781428656,0.11640351319111848,4.664020110586662,0.0018239324882582677},{3.5792846199317045,0.000008056487000841957,0.008807474026397921,0.005750959201527773,0.36747734551724387,3.1044295291855493,0.0012440628116487948},{6.635882648882845,0.000012810045663998252,0.006406130571414215,0.00825799385117243,0.23340337294389768,2.380876665429791,0.0009479683902985885},{5.631264516518526,0.000006483056520870152,0.012298795111764661,0.006516844737844397,0.11915005914685893,1.8217301937940826,0.0020143531657058266},{8.56781209080961,0.000011794880159375573,0.008244518669529466,0.007202134738539945,0.17686196859298864,1.590637753918613,0.0018261340989639701},{5.700626613135666,0.000006512465843389875,0.014772664937974423,0.005771495825570492,0.13186711374601115,1.5696940409568665,0.002045258146204537},{4.487912732585145,0.000015515741509729788,0.005296851954969251,0.003105695457535651,0.2812486429894639,3.561302558672037,0.0014654357985925094},{4.4777948283259255,0.00001012375681564577,0.0058841823575052745,0.008725982834589257,0.15305213246552826,4.089431189709945,0.0016532169675213826},{2.411905383541706,0.000015162674926823146,0.012825635120968971,0.00589020877823967,0.08513463482395123,4.20935202839412,0.0008280721949028052},{5.326300159127798,0.00001369068681639699,0.005018689021573009,0.0031665680014004403,0.3929527253614876,3.0831740123553155,0.001606337037790346},{7.7867196160841035,0.000014884234222850196,0.0077206062435784435,0.0055561893773379365,0.08301606848546239,1.8731801619895636,0.0012309517156049456},{3.5593391419818547,0.00001059212410050904,0.012887976167951232,0.004817431904227716,0.1142864252783049,2.6230881325028985,0.0015938786277133882},{8.884315373746604,0.000005781030027969028,0.010398666189527672,0.011131515081779942,0.0953383917597702,1.5288050622523226,0.001529725051728342},{8.707819560365543,0.000021138257935459324,0.007574533651821369,0.0021747599505218497,0.1943079408416801,1.412420583602735,0.0018925251570172472},{5.425589369729392,0.0000137537827080698,0.009938531202529834,0.0035245107292589764,0.11911569201935578,1.8366243888274525,0.001334046175983234},{7.405959903473939,0.000016733347605680846,0.006866353046796326,0.0021655719748785085,0.09725992302131431,1.7330924620156711,0.0017889219061811925},{5.1735045480825805,0.000012923269130196166,0.005231573380218624,0.010158957112212753,0.12247367688441035,4.103632370325924,0.0014888253353921833},{6.394831394774266,0.000023910481546724035,0.006698147428031387,0.005163701481755645,0.10365103522638988,2.286485972658239,0.0008315032103812387},{2.9784795633934125,0.000030036159492153722,0.007542606552029119,0.0032993107260308376,0.10957858820116968,4.492093688830256,0.0013231549622962286},{7.392442395841507,0.00002363973849531992,0.00905880955446583,0.0021777010697330203,0.11449353633732207,1.5522247864824772,0.0018950250158353379},{6.707264977397192,0.00002382467333928828,0.00823798540556724,0.0034899871118206516,0.1004551400091077,1.7906683992690562,0.0011343976128122258},{5.130714083384829,0.000016917096592274402,0.008263569094226143,0.002822777092020461,0.23175602132399434,2.0595867791751834,0.0011864178778014387},{2.1545213415834774,0.00001621958339821636,0.012065781109261355,0.0038413900238505596,0.16251223807727558,4.376821466209391,0.0015158173956426073}};**

CFFM1

**Table={r_1, K_1, k_1, q_1, w^(2)_1, ,r_3, K_3, k_3, q_3, w^(3)_1}**

**w^(2)_{-1}= w^(3)_{-1}=1, \tilde{w}^(3)_{-1}=1/10.**

**{{1.7771778480712346`, 1.880194627154487`, 0.015746746993775373`,**

**0.03808061200889032`, 0.000265148996133259`, 8.835307035478714`,**

**4.682148567956235`, 0.01050501440532698`, 0.032699140884150824`,**

**0.004662053690803008`}, {1.101294803184345`, 2.713479536246462`,**

**0.0334479349106854`, 0.006375288277185892`,**

**0.0002034456904477771`, 8.749069940988338`, 3.9079779239159196`,**

**0.015398071188581296`, 0.021032785242886896`,**

**0.0022205293758030334`}, {1.088022689419553`, 4.813106674113354`,**

**0.015517809849061447`, 0.02157764694547376`,**

**0.00011797408640442514`, 6.90451582148717`, 4.73283855134709`,**

**0.013992935831220778`, 0.02015439427362884`,**

**0.005268353804245014`}, {4.374365889985089`, 1.0212251976921154`,**

**0.04481009759675629`, 0.01998295756873815`,**

**0.0002725110285280042`, 7.093182054785212`, 3.552396562333816`,**

**0.014944608702135265`, 0.013601523327230823`,**

**0.006411802560614141`}, {1.0814170475567941`, 2.455571850912513`,**

**0.015997805058269518`, 0.020542474422059365`,**

**0.00030055167566724744`, 9.768292317532676`, 2.6649252107995185`,**

**0.012810534104253216`, 0.015957903663669115`,**

**0.0005367235557627078`}, {1.2454232993198406`,**

**1.5313682310107062`, 0.03256660072570486`, 0.005656383472504606`,**

**0.0003342493445264317`, 8.878904717306341`, 4.656707962960168`,**

**0.021732906068016998`, 0.019983568016863518`,**

**0.0013646019211337873`}, {2.3718591803095617`, 4.39268149102699`,**

**0.031954883591221325`, 0.03353886745169485`,**

**0.00011834407691847099`, 9.757075041180705`, 2.8285785514966832`,**

**0.008027327198809268`, 0.022161182761067356`,**

**0.005544730203084396`}, {1.7367017043857977`, 2.6443410779729817`,**

**0.009280438716773108`, 0.019638283702033375`,**

**0.0001486832254062563`, 7.94289267836206`, 4.2164411246612685`,**

**0.020633938392892764`, 0.018070104339066327`,**

**0.006396379686326744`}, {2.271631413787242`, 1.8673931322965815`,**

**0.0433441365359426`, 0.018707151505465064`,**

**0.0002693532934574805`, 9.069147670675083`, 2.4923335126699664`,**

**0.008917905413537333`, 0.01720979393108024`,**

**0.003505348197215456`}, {2.236086402618854`, 1.8448582902360648`,**

**0.02481833876434142`, 0.020274925077963926`,**

**0.00018145785863421526`, 9.934529165648492`, 4.574982510191114`,**

**0.03223200331865124`, 0.012894414374488149`,**

**0.00573927559280297`}, {1.1920263927095949`, 4.230939842667669`,**

**0.006190778196446291`, 0.047223347466095175`,**

**0.000157165825685604`, 9.089290924821228`, 4.374422712540577`,**

**0.011326489045009026`, 0.031585945879070404`,**

**0.007066144995597342`}, {3.1040882416332316`, 1.98780289192002`,**

**0.02997659618861083`, 0.03835063766959594`,**

**0.0001381786202366113`, 8.933343195962415`, 4.8094359061826575`,**

**0.01115108997514666`, 0.021043137105515204`,**

**0.003095247857914825`}, {1.5695489899274353`, 3.968349886554835`,**

**0.009954388369840993`, 0.0408671708175508`,**

**0.00013863383932565324`, 6.946788064570184`, 4.932979196183092`,**

**0.030125143068542776`, 0.010294200312060658`,**

**0.00878828882800666`}, {9.489356627485819`, 1.1910821773134517`,**

**0.023506923288513906`, 0.03972782044174428`,**

**0.00011691205875184914`, 6.774123524005745`, 3.495920711739907`,**

**0.008723358412011828`, 0.020117126838987046`,**

**0.002426927060660937`}, {1.9006063583782282`, 3.079875413278347`,**

**0.025570505484086695`, 0.043866200447131354`,**

**0.0001741160848545524`, 7.750828341933397`, 4.203923020834312`,**

**0.009363856233029662`, 0.020418037094476607`,**

**0.0008010568391443214`}, {1.5437471890976866`,**

**2.7319203748191114`, 0.0415528818330238`, 0.015980597116785712`,**

**0.00023233989409122448`, 9.905089551840323`, 3.9040424866226084`,**

**0.03107100479664858`, 0.011228825997779433`,**

**0.00992023257116262`}, {4.512876974857871`, 2.5728936367978443`,**

**0.019990958208323487`, 0.029581469867201754`,**

**0.00010176144103142375`, 7.129199683793601`, 3.1096631737451004`,**

**0.014467760592081574`, 0.013820648353212346`,**

**0.009655252046068001`}, {3.784542983701307`, 1.1526377153708438`,**

**0.034180996647143316`, 0.008460458518078348`,**

**0.00019127362721845127`, 6.592567738666521`, 4.849698329990599`,**

**0.029116357974956125`, 0.010342066810458676`,**

**0.0008584994375295044`}, {3.2234481513561413`,**

**1.3026946405718993`, 0.035799589064354154`, 0.038030376548451386`,**

**0.0003197508791553238`, 7.535598198600617`, 3.5197759226936363`,**

**0.017779036757108783`, 0.008703270892883523`,**

**0.005693765513811077`}, {2.809162576439576`, 2.7749688797864476`,**

**0.047234343285052785`, 0.014834101509647556`,**

**0.00012523720351216051`, 8.58924148769201`, 3.8439615136289493`,**

**0.00979311698672889`, 0.02570224532576336`,**

**0.006998047693057148`}, {1.9778735032517485`, 1.3659559092665399`,**

**0.04980065717501028`, 0.005175364717163594`,**

**0.0003273055574764764`, 9.904005077207405`, 3.7067726598281405`,**

**0.024839758652407895`, 0.015022706249644226`,**

**0.008039169001567844`}, {4.315545875175832`, 2.250528369196071`,**

**0.035240643218372544`, 0.0275494809587222`,**

**0.00013247090366199157`, 8.285598493788104`, 2.8582655378524313`,**

**0.01588912683817248`, 0.01149321032090854`,**

**0.0030227033633506827`}, {1.2972730318562515`,**

**3.2428511841450707`, 0.043511143376147615`,**

**0.0054804366888477965`, 0.0001774237873064504`,**

**9.168932308888035`, 4.5076720172372475`, 0.011187220902564195`,**

**0.033790163565029396`,**

**0.0004394880145806475`}, {1.5324896290636474`,**

**1.7897744609882578`, 0.012846858583522282`, 0.04336246748306244`,**

**0.0003507611447972122`, 8.683392293726044`, 3.8855947394935093`,**

**0.015548962164029553`, 0.020358295063488754`,**

**0.005055719258400138`}, {2.1739438443514256`, 1.605093247328612`,**

**0.04234700198840266`, 0.024442100433184635`,**

**0.0003429866763541567`, 8.63136909316854`, 3.513075521885952`,**

**0.016119439815310582`, 0.015458488109077954`,**

**0.0038836181261850677`}, {1.0592786020194147`,**

**3.9819402961116355`, 0.03872178186724755`, 0.006618572883746399`,**

**0.00016832907436316324`, 7.000055197207532`, 4.822520597764366`,**

**0.01612134239282719`, 0.018359000520620262`,**

**0.005411925941706747`}, {1.505591262751011`, 2.116277630987775`,**

**0.04161745401290015`, 0.0055030946724383065`,**

**0.00027481843361465766`, 8.723875424812231`, 2.6281709846235453`,**

**0.011977268548161182`, 0.015470118412418188`,**

**0.005947036376549265`}, {1.1867921042478038`, 3.4770626691430397`,**

**0.02443685665769857`, 0.029630954080010236`,**

**0.0002208164258334349`, 9.594167983458775`, 3.823334046744365`,**

**0.01904301410194669`, 0.016675911066369495`,**

**0.00020169711856958365`}, {1.3062329988211498`,**

**3.6399643188210202`, 0.021010884996779118`, 0.0127047268767825`,**

**0.0001826405649002459`, 6.65047647498297`, 3.863299684895667`,**

**0.02424787674951749`, 0.0097815388948361`,**

**0.007351157147397357`}, {1.8629719956246547`, 1.8333069570767364`,**

**0.010727669522862235`, 0.031077137643603286`,**

**0.0001816904372398051`, 7.510157792553219`, 4.639873330731205`,**

**0.02024754899128569`, 0.014687553792413995`,**

**0.0041014603250679165`}, {1.3448883088195167`, 1.51600583776891`,**

**0.009275577847214514`, 0.03188827863336534`,**

**0.0004780381739180224`, 8.584425081870972`, 2.629560392216457`,**

**0.008930645910310883`, 0.020800924082252828`,**

**0.00963472056260325`}, {4.65070218629398`, 2.4760010746593872`,**

**0.044939543486353525`, 0.018062770376405968`,**

**0.00010701681919633721`, 7.517268132577225`, 3.4642037553906615`,**

**0.023253018708429926`, 0.008555159456992978`,**

**0.005270450896678887`}, {3.847016851866332`, 1.2417377649254249`,**

**0.03202886103373599`, 0.03644881702277927`,**

**0.00024240799306201807`, 9.594184316357332`, 3.07529312313069`,**

**0.014015437057582937`, 0.014293706522842821`,**

**0.005522968141411004`}, {2.027950004710732`, 1.507949555848561`,**

**0.029121393606014484`, 0.012160115211213872`,**

**0.00020735604762648664`, 8.440353727235877`, 3.8005468775307563`,**

**0.017124653858170205`, 0.015226009257454091`,**

**0.0015551972373795183`}, {2.7195791457686003`,**

**2.4401433599635833`, 0.012284921390893405`, 0.014372852678938931`,**

**0.00011163703131711854`, 5.973831128885536`, 4.0023750936707545`,**

**0.024293794224428317`, 0.0085127638348571`,**

**0.007669124446555124`}, {2.1830395565649905`, 2.267783270967713`,**

**0.018172310621366677`, 0.021865676095878674`,**

**0.00014401871977376933`, 7.244292544697055`, 3.6690392816175565`,**

**0.015015048824378562`, 0.014155270179896172`,**

**0.0004351281535127458`}, {2.2650454306573575`,**

**1.0252171698472736`, 0.023700032683560857`, 0.008240484430398494`,**

**0.00025863281255743276`, 7.863805037001567`, 4.628057898117719`,**

**0.033783319753499366`, 0.009975170323079874`,**

**0.0020514698465208744`}, {2.896095442185697`, 1.2183038205287877`,**

**0.010407891263126502`, 0.007885122614339032`,**

**0.00016564836199447`, 7.512222366909539`, 4.016217321251809`,**

**0.019353737398148968`, 0.01682135118786808`,**

**0.004105411542637941`}, {2.7669707755194324`, 1.142923990570556`,**

**0.04494458066894989`, 0.029859568106747073`,**

**0.0003368854588845088`, 7.676110878606281`, 4.932208517467086`,**

**0.02355785466335182`, 0.008120002294075175`,**

**0.008471780778431573`}, {1.0492265861490573`, 2.0019131873314926`,**

**0.005316570447601178`, 0.04200064615628615`,**

**0.0004687663461841896`, 3.9937212002702207`, 4.819714661507024`,**

**0.012538505424570058`, 0.012297594022747084`,**

**0.0068684173641838705`}, {2.0697156093795837`, 1.123316722484942`,**

**0.015722272846320856`, 0.036204396499564`,**

**0.0002832487427566685`, 9.283160096951075`, 4.950852159524326`,**

**0.022023904189629948`, 0.01318613162797607`,**

**0.00599511234913336`}, {1.6585862123702455`, 1.9970221860833943`,**

**0.04297230937788615`, 0.012879996994198006`,**

**0.0003238921784223577`, 8.642783411688068`, 2.834788418502776`,**

**0.011242968053407382`, 0.017520403356909776`,**

**0.006626966795953952`}, {3.4043112083878153`, 3.4792639061541157`,**

**0.04994353220936448`, 0.008048375687088168`,**

**0.00010662028929531338`, 5.626104730289709`, 3.6423161968606754`,**

**0.01252511937887233`, 0.013617999851730594`,**

**0.0071487123994384855`}, {1.4484496941043137`, 4.646398627141731`,**

**0.03228107508978298`, 0.023844531309467426`,**

**0.000145005954843647`, 7.398143375982563`, 4.551936401731203`,**

**0.02931860135194353`, 0.008481267843796686`,**

**0.007492523986834509`}, {2.9044125944823485`, 1.3736106193783417`,**

**0.014986114605254716`, 0.02875197228846489`,**

**0.00021596103689082782`, 8.348388164364419`, 3.6952664343426296`,**

**0.022539719381687806`, 0.01329808801753931`,**

**0.00045091325243658796`}, {3.6500661366068066`,**

**1.1078953817700077`, 0.013062710505094922`, 0.020697392981312096`,**

**0.00018289583976731014`, 8.600078654221921`, 4.012994217479979`,**

**0.00636847953701674`, 0.041100887416672394`,**

**0.0032075753983762754`}, {1.2151543576720627`, 3.703681008335728`,**

**0.046004210891285896`, 0.0062866433669522895`,**

**0.00023200810537114987`, 6.615127896348673`, 4.285882242929396`,**

**0.010209346343077567`, 0.02654810894901448`,**

**0.0003247129531819097`}, {1.3113880503042754`,**

**1.1944641014889816`, 0.006953973890620788`, 0.005372813343363328`,**

**0.0003571329624476073`, 5.1112680942556565`, 4.883082714996411`,**

**0.021933935280684907`, 0.009649341207173555`,**

**0.009941066331926504`}, {1.817589196056371`, 3.304352140305837`,**

**0.042319978310884285`, 0.01063112564623065`,**

**0.00012084957383218589`, 9.881389091404312`, 4.712685900852152`,**

**0.025243621592490828`, 0.017795326728663075`,**

**0.009253947981460722`}, {3.0759218342979793`, 2.3547520825977992`,**

**0.037718284649860226`, 0.014106387806445131`,**

**0.00010998884884941273`, 9.530949014454503`, 4.007500725517265`,**

**0.013897248172908404`, 0.023696317036367154`,**

**0.001900908708700971`}};**

CFFM2

**Table={r_1, K_1, k_1, q_1, w^(2)_1, ,r_3, K_3, k_3, q_3, w^(3)_1, a, b}**

**w^(2)_{-1}= w^(3)_{-1}=1.**

**{{1.553861758380469`, 1.3034802756322303`,**

**0.005395854218433693`, 0.04938779338862481`,**

**0.0024241196672121455`, 5.391630820953907`, 4.859007074230855`,**

**0.01878703405855907`, 0.012457541797449553`,**

**0.00002973199558075849`,**

**0.07636448572471674`}, {4.196158984954971`, 1.0118922225018014`,**

**0.016854095125321655`, 0.017275200135107756`,**

**0.0009676477896138225`, 6.97417823654353`, 3.9999326695761868`,**

**0.01766555208633918`, 0.013100950960787054`,**

**7.055948876075477`*^-6,**

**0.0517733206717193`}, {2.3537515618084406`, 1.5292739217404483`,**

**0.02844673926172664`, 0.020936846946033816`,**

**0.001218172411892305`, 6.694941502775309`, 4.835693367274133`,**

**0.020067261823972088`, 0.012688907723047121`,**

**9.515263875194833`*^-6,**

**0.054908363392818016`}, {2.2677575096275824`, 1.1823046755068818`,**

**0.04057674955040513`, 0.025519573706870885`,**

**0.0013917123743037892`, 9.467190234154895`, 3.7716976344651583`,**

**0.011433128101409548`, 0.024017320385320465`,**

**0.000029970859633699035`,**

**0.021668600864555562`}, {1.3768395476935904`, 2.578274263730237`,**

**0.04016960210618031`, 0.019087129201918795`,**

**0.0008918636617152916`, 8.76625102324779`, 4.6360313135481235`,**

**0.011760872468052574`, 0.02601633533183287`,**

**0.000022675401553178257`,**

**0.0560226681802031`}, {1.261105214541777`, 1.8693367622223587`,**

**0.03579484839080449`, 0.02884963500527997`,**

**0.0020969046900000676`, 9.75001088220008`, 4.880866573570753`,**

**0.03436323282481188`, 0.010554106573101248`,**

**0.000011083588013191045`,**

**0.012765650530188216`}, {1.0921182319143163`, 1.6688064458332246`,**

**0.03467155508222736`, 0.022402247932064814`,**

**0.002913475111278263`, 6.239616711227157`, 4.222500594778297`,**

**0.010118899350855672`, 0.01889937820591546`,**

**0.00002819569483899912`,**

**0.05856089421600397`}, {3.6560269815603874`, 1.6471943249729275`,**

**0.014278689219903488`, 0.03696738197706537`,**

**0.00043792238920429064`, 9.980559538157202`, 3.8431976038462734`,**

**0.009509154298871461`, 0.03186156509110258`,**

**0.000017642945404484175`,**

**0.06524567976178319`}, {1.06339727268149`, 3.4078406236604524`,**

**0.011717616575981027`, 0.042734657267621365`,**

**0.0010132849993379923`, 6.844343349992265`, 4.32125501980383`,**

**0.012618433900372657`, 0.02004640812821848`,**

**0.000023608364388630104`,**

**0.07866828092004521`}, {1.1681890770105205`, 1.015563122602411`,**

**0.029550846895034927`, 0.009169328991810277`,**

**0.005884835898172932`, 5.981211513920336`, 4.6411226755565895`,**

**0.012862141646789761`, 0.019634318162329877`,**

**0.000011639036893448354`,**

**0.04607464131872471`}, {1.3081768812267445`, 2.3433624201887744`,**

**0.03395699539328437`, 0.025480612005855247`,**

**0.0016326196559034063`, 8.099956044627948`, 3.5064368200769884`,**

**0.01713948267874403`, 0.011155912016453752`,**

**0.000012636876758397538`,**

**0.04714764868776189`}, {2.093775036118476`, 1.0143640651856307`,**

**0.034064689441477`, 0.026154140160297268`, 0.004088443695178431`,**

**8.434164498802957`, 2.753955365845922`, 0.0159164913142522`,**

**0.011166389802420545`, 0.00001950813420140781`,**

**0.05754217198949918`}, {8.376407787126912`, 1.297260233654952`,**

**0.02966048028479737`, 0.04372013010701124`,**

**0.00021649727175383597`, 9.711634698684747`, 4.764021936527056`,**

**0.021065807786048982`, 0.012806451740816654`,**

**0.000022993249127801737`,**

**0.0995043185517995`}, {1.3806602650454547`, 1.5165331185928883`,**

**0.04462246938958171`, 0.04252610883032569`,**

**0.0020993113846812326`, 9.799492565603188`, 4.944055998084988`,**

**0.013876933920361247`, 0.013822820889339463`,**

**0.000026392121165916636`,**

**0.05122608932197634`}, {1.7265983646170078`, 1.147965361158355`,**

**0.025864551046043155`, 0.0347008271608029`, 0.004996335402177225`,**

**7.933316507741056`, 3.4179900692954224`, 0.014136932192926027`,**

**0.014267324949367333`, 6.9476941435763585`*^-6,**

**0.01868547482316439`}, {1.9539540126816348`, 1.330183866381895`,**

**0.018762091858081593`, 0.018331217167410006`,**

**0.001114355936166142`, 6.3305273720583095`, 4.449392447398128`,**

**0.015406056102232911`, 0.016621320670550485`,**

**0.000028052492160378276`,**

**0.06516485281441906`}, {1.0309037545352506`, 1.0826504245897812`,**

**0.015769371497789543`, 0.02859285907600631`,**

**0.007363416885631165`, 8.415455379406772`, 2.82329162004088`,**

**0.015019538483624695`, 0.012315220423037301`,**

**0.00002130093144315524`,**

**0.07980075021877747`}, {1.0270641104292153`, 2.1512438183738336`,**

**0.018153571911651144`, 0.022936021332252213`,**

**0.0012807485017154521`, 7.780682695369629`, 4.017171969049114`,**

**0.025164653623422545`, 0.011348263495980344`,**

**0.0000372264744039415`,**

**0.04190090844840533`}, {1.4538303981546719`, 2.605789491438376`,**

**0.03601495523892079`, 0.009537700702935296`,**

**0.001196871098897715`, 8.071621484893281`, 3.5147785845263204`,**

**0.022069475938115507`, 0.010662725028397331`,**

**6.988396854955488`*^-6,**

**0.03658988023393113`}, {1.1610837174586752`, 1.583929023286415`,**

**0.044109051373451696`, 0.03343870745178798`,**

**0.005505497859413611`, 7.648260473563759`, 3.7504193673845263`,**

**0.020602792126770887`, 0.00983901460145279`,**

**0.0000138576534880396`,**

**0.018627834676809285`}, {1.129817239878502`, 2.5049780284174528`,**

**0.02148199670325214`, 0.01639400690876841`,**

**0.0021345529384102866`, 6.049334045412699`, 4.528622389143168`,**

**0.02743793761881115`, 0.008050043019311205`,**

**0.000011881693505452525`,**

**0.07828795397959484`}, {2.251031696896531`, 1.5361431268665129`,**

**0.02506056097182449`, 0.029996001314070098`,**

**0.001630694654115002`, 5.585857508546695`, 3.6853922696321035`,**

**0.010210356500061898`, 0.014880413986931441`,**

**0.000032171006677407014`,**

**0.09873215747788311`}, {1.279359260189695`, 2.776826697094399`,**

**0.023996115036599486`, 0.011330333244181458`,**

**0.000916656484543088`, 7.57709863102103`, 4.457373434889579`,**

**0.021654594686585364`, 0.013485464618460612`,**

**5.966302887232992`*^-6,**

**0.04226555432308818`}, {1.213791474530769`, 1.4530316688718363`,**

**0.02467721331914463`, 0.030181214363773384`,**

**0.0063448122364002605`, 9.305358809547041`, 3.363606848453184`,**

**0.012522214600335188`, 0.01966263795458395`,**

**5.8742357127762205`*^-6,**

**0.03986537952899849`}, {1.3295039972272065`, 1.0940207383217206`,**

**0.031085928223248377`, 0.016409958678153064`,**

**0.006575512404360153`, 9.64699337406892`, 3.099810924072961`,**

**0.009119843623531548`, 0.022497496080836274`,**

**8.845612087036086`*^-6,**

**0.08816021758235473`}, {1.8072482826403764`, 1.009015375663286`,**

**0.019768235912649368`, 0.02122037144080436`,**

**0.0031061939834918385`, 9.898173479573849`, 4.320845939692986`,**

**0.03922526733360006`, 0.00929284876681042`,**

**0.00001171569116416045`,**

**0.07167434060635358`}, {1.0349941516995038`, 2.4484759271639573`,**

**0.02359930788135342`, 0.04271417881473899`,**

**0.0026458409782543057`, 7.95083460234704`, 3.888377104821741`,**

**0.018160995213624763`, 0.0110515588743407`,**

**0.000014722204711538667`,**

**0.06124116626258691`}, {1.0540083061183196`, 2.1584704936646943`,**

**0.014054640889117578`, 0.04137861627557521`, 0.00310862745304466`,**

**9.876281704689912`, 2.419892101778733`, 0.010765842589378953`,**

**0.01780687413231031`, 0.000021078354581154752`,**

**0.09276404451245038`}, {2.086334793960276`, 1.1785212097921969`,**

**0.025083059205818173`, 0.0403125347301584`,**

**0.0018663187847266314`, 9.48119573649469`, 4.568293354334004`,**

**0.02199204596995661`, 0.01233067499250412`,**

**0.000013306800349831227`,**

**0.05529633520138455`}, {1.9033313055813696`, 1.2333075535744022`,**

**0.034121860653636316`, 0.029957783632084248`,**

**0.00628614452121855`, 9.638946776228437`, 2.399084850526691`,**

**0.011972631125900988`, 0.012845385840152435`,**

**6.243471737333557`*^-6,**

**0.050694486436412084`}, {2.1374114789213365`, 3.9459719670932287`,**

**0.019845931916815565`, 0.04350152308133244`,**

**0.00026433035300486504`, 9.276845575383835`, 4.871313057278595`,**

**0.017504974238017806`, 0.01872151807214157`,**

**0.000011758038305555395`,**

**0.03182185308360433`}, {1.1349702309937175`, 2.4314787552437434`,**

**0.010425654400885248`, 0.03889664357233613`,**

**0.000999507045269122`, 7.669051996439144`, 4.643723196160088`,**

**0.020677113224658462`, 0.01349349236507933`,**

**0.000018628416678999523`,**

**0.07269896983971766`}, {1.4629431393822347`, 3.4399972140451682`,**

**0.045727518987516785`, 0.044967979459082055`,**

**0.000999205206442113`, 9.700070625070488`, 4.162889997484762`,**

**0.017961914195758412`, 0.01674438952014285`,**

**0.000019661546569280253`,**

**0.027743605842354066`}, {2.4265922973147482`, 2.9888480564603714`,**

**0.04332552529618609`, 0.03130920359270896`,**

**0.0009053869618530237`, 5.664418908919167`, 4.648487738756704`,**

**0.013702614233414102`, 0.012519803255607878`,**

**0.000011855733325632166`,**

**0.0779128652479722`}, {1.1124469980681084`, 1.3874155407873774`,**

**0.011548719843941554`, 0.039859899146462804`,**

**0.003471811963288815`, 7.050653641395332`, 4.147237304640293`,**

**0.016051535573046095`, 0.014487248179185064`,**

**0.000019068892531689074`,**

**0.07846887813860268`}, {1.1035465414561791`, 1.716552094417171`,**

**0.04176593875060185`, 0.02992789639501367`, 0.004023796826790934`,**

**7.593752165887727`, 3.7936599873613304`, 0.012937750184424529`,**

**0.016598751878200202`, 0.00002927398084044161`,**

**0.061092892445275104`}, {1.7407094823948448`, 1.1376743916671375`,**

**0.01243029033682952`, 0.013885025968422567`,**

**0.001977926770523374`, 8.273788704801994`, 4.5159720228910905`,**

**0.014138771980282683`, 0.028828819799600294`,**

**0.000011673165512786265`,**

**0.08827206135125376`}, {1.9671313200255423`, 1.2905121335351781`,**

**0.027927672998957057`, 0.020591738976862735`,**

**0.002893191148203328`, 8.77477919370991`, 3.9224282476699814`,**

**0.019621641361542326`, 0.015111960126089452`,**

**5.909005469404749`*^-6,**

**0.04988825528825727`}, {1.5581022454402031`, 1.006444284091459`,**

**0.04292563894814075`, 0.019280287588716055`,**

**0.0033311990970161173`, 9.853777178394353`, 2.815837632815528`,**

**0.0183032305718517`, 0.011890717685090292`,**

**0.00002885811642046367`,**

**0.017959894120983075`}, {1.0153900870611938`, 1.2042890371220834`,**

**0.03290820505556652`, 0.025514479811214907`,**

**0.009882024976938452`, 6.362395456617691`, 4.738596024945269`,**

**0.014388208406511921`, 0.012455483256843279`,**

**8.328067804303749`*^-6,**

**0.0632003662343274`}, {1.2529867800199916`, 1.594219697886273`,**

**0.029427278734800028`, 0.008607538492942234`,**

**0.001496758420653506`, 9.683775491312318`, 4.67006617153159`,**

**0.014294724641452365`, 0.02474814357435097`,**

**9.75587515096487`*^-6,**

**0.027915966016473323`}, {1.1328329306022304`, 2.1802250442754003`,**

**0.015832618839653687`, 0.03304712266416454`,**

**0.0009364094521595173`, 9.478997776794092`, 4.552961036372308`,**

**0.023389110143292577`, 0.01678091147373542`,**

**0.00003657575048977142`,**

**0.06984496956424058`}, {1.7031770919079676`, 1.9478323832732922`,**

**0.010361428461290186`, 0.04981421857748118`,**

**0.0017543681218496563`, 8.601036934939955`, 4.2855131985951544`,**

**0.027838775915007136`, 0.010913801176431391`,**

**0.000012130677526459443`,**

**0.09996287570989071`}, {2.6943692968713933`, 1.7067144640317737`,**

**0.040041689878343834`, 0.020553602351416435`,**

**0.0008812628940558353`, 9.548976488112704`, 3.8133054677903413`,**

**0.026769789659629492`, 0.011657130454247829`,**

**9.692406947284879`*^-6,**

**0.01607467422614374`}, {1.0503578496598251`, 1.0033441922070754`,**

**0.01060999695657245`, 0.01168513896998502`,**

**0.0032606782828123234`, 6.012807937707864`, 4.941108298749838`,**

**0.033527919090362956`, 0.007188256845432207`,**

**0.000030059399067831038`,**

**0.07868803757315157`}, {2.549434643159678`, 1.0917677041649112`,**

**0.03040505581705727`, 0.041355279672750475`,**

**0.0020007853863696634`, 9.432105387284437`, 4.260087546187759`,**

**0.013458357315851002`, 0.019167987733451075`,**

**0.00001943616207278701`,**

**0.08017675846862213`}, {4.362439894978619`, 1.102418703184731`,**

**0.025025043628252072`, 0.020892279457320827`,**

**0.0006261580942142544`, 8.571368144315795`, 4.851859810053856`,**

**0.02214658838703113`, 0.01750213336962468`,**

**0.000017296349483706364`,**

**0.09316927067467667`}, {1.3145713664861098`, 1.9524591669001836`,**

**0.03553778665337448`, 0.008874001903033946`,**

**0.0014548378798192527`, 8.15367643381655`, 4.613601274375242`,**

**0.02669699322681282`, 0.013174233960678669`,**

**0.00001832200765309212`,**

**0.0752235203509049`}, {3.497777621637484`, 1.4512243207084499`,**

**0.008619271297047655`, 0.04992227689274106`,**

**0.0008018019762441563`, 7.450856841596606`, 4.889324129360032`,**

**0.01143025065531212`, 0.024754375355466998`,**

**9.38262119158645`*^-6,**

**0.06032862258553859`}, {1.2117916518716747`, 3.3755027145028267`,**

**0.03724926199795152`, 0.022475144540923482`,**

**0.0008664277399350745`, 9.846307681640127`, 3.8466245872524`,**

**0.017347785022278565`, 0.015063198547311876`,**

**0.00001526783969949663`, 0.06374941825405564`}};**

CFFM1(-)

**Table={r_1, K_1, k_1, q_1, w^(1)_1, ,r_3, K_3, k_3, q_3, w^(3)_1}**

**w^(1)_{-1}= w^(3)_{-1}=1, \tilde{w}^(1)_{-1}=1/10.**

**{{4.544661959188327`,3.9215799188588187`,0.031282465570812865`,0.010445015401819923`,0.000041951357104545945`,5.4797533329989285`,4.473191658582358`,0.017458002422660213`,0.002372542981674823`,0.000041951357104545945`},{4.813963669535489`,2.6878390702461115`,0.04784398103169607`,0.008527003215624912`,0.00005481080282840377`,8.535740682933529`,4.0969427486161445`,0.011239286026542637`,0.003442594413234478`,0.00005481080282840377`},{1.9856998900101326`,3.421936637595979`,0.00910536307717006`,0.016422160238061773`,0.00008488924772416073`,4.864900561465612`,4.548620771285059`,0.01227795281818285`,0.002057087317780263`,0.00008488924772416073`},{3.9558777726756036`,1.5349404237946018`,0.016595709664683878`,0.006729351588862391`,0.00006668279841281349`,9.318192106520023`,4.182007829665654`,0.013745472436344462`,0.005602957209054814`,0.00006668279841281349`},{3.31394587970878`,3.710877604249591`,0.03245525919633786`,0.007614298119350368`,0.00004041503170978803`,8.130018818757193`,4.7272671504164805`,0.009364480429661183`,0.00932060415848195`,0.00004041503170978803`},{1.430922844169718`,4.3092237056578675`,0.014699161206135292`,0.01041164910918526`,0.00007729724360603231`,8.419619924798809`,4.313732800647492`,0.014024524675958036`,0.0040638955085573365`,0.00007729724360603231`},{3.902532753473663`,1.723299665245019`,0.009342683275921401`,0.014410132155874868`,0.00008552619535960788`,7.363206751242192`,2.593064978420273`,0.007104937917399981`,0.004941343533915385`,0.00008552619535960788`},{1.658451831510126`,4.794145846498787`,0.007116408171109166`,0.01979544204574481`,0.000059040927267997416`,8.608329691950384`,3.8762078957616977`,0.00936565349737279`,0.010585236012102085`,0.000059040927267997416`},{3.0911753350712914`,4.682083448857498`,0.0287623779025883`,0.009060723389490138`,0.00005694953946626456`,6.778948455696742`,2.320790112102072`,0.006592479849533803`,0.0053128254889682446`,0.00005694953946626456`},{2.279107267489101`,3.401240752886536`,0.030235902095924777`,0.00720674050365697`,0.00007869647181150247`,9.11319139475816`,3.6632715839962513`,0.01115092141666365`,0.003798176018957121`,0.00007869647181150247`},{4.358622501987337`,1.8918097173060673`,0.015006937722148894`,0.010027101744278562`,0.000057868578395975194`,7.2049048600500125`,4.363664957189802`,0.016083198665934176`,0.005531788902515586`,0.000057868578395975194`},{4.0087844082158215`,2.223594398838036`,0.023413319580443306`,0.01123633242415914`,0.00005209200565626615`,8.546705062982447`,4.602575876736388`,0.009309079800120489`,0.006937475914183561`,0.00005209200565626615`},{3.1627425445135815`,4.327192898507608`,0.015626416150728496`,0.019764264075823188`,0.00004412300372886799`,8.194187888741066`,3.494027620961771`,0.00614788131186058`,0.012245389413438699`,0.00004412300372886799`},{1.387132687019177`,4.873289143536332`,0.01561874727053874`,0.010212509577446637`,0.00008844601417049838`,7.678234310510874`,3.4659755246257786`,0.015120220295430295`,0.0023965139229382253`,0.00008844601417049838`},{3.6333424882807206`,2.8731814250415884`,0.019603571366051002`,0.018413836337117327`,0.0000768320713184379`,9.094826222084848`,2.1519287934996534`,0.005825028541610933`,0.003711090097038787`,0.0000768320713184379`},{3.0253952805100743`,4.442798147129934`,0.02498832070754728`,0.009640294801189869`,0.00004687312770776343`,9.006568056512513`,3.2994232540942177`,0.014597159608394819`,0.007907294727268678`,0.00004687312770776343`},{2.8780326161810743`,2.9675594594096353`,0.049007445841564745`,0.0073758548591411725`,0.00008246415499192378`,8.92830843978913`,4.195625388027344`,0.006329931436502845`,0.004796321360169609`,0.00008246415499192378`},{1.5253972251334513`,4.955753668248468`,0.015107681350681322`,0.012012590316497252`,0.0001093316843921385`,6.486843129522333`,2.4075257872513527`,0.005277018816759453`,0.0048030609386506655`,0.0001093316843921385`},{1.9270237431428479`,3.1045655038155715`,0.018758425645706213`,0.010899394035091076`,0.00006786777706314939`,8.982918978567419`,4.29294772505439`,0.007318732977368125`,0.005377725011184675`,0.00006786777706314939`},{3.674164758119426`,4.761159763861317`,0.03585988980353859`,0.01337485035787105`,0.000030229608746695953`,9.894696741573913`,4.992152773523277`,0.019129029667539543`,0.0027701903907649816`,0.000030229608746695953`},{2.0819376849439726`,1.4665817589800856`,0.010806422964787621`,0.011572378591854948`,0.00014106820275762628`,9.23027739030898`,3.94699180736649`,0.005886766302556114`,0.002108996829927035`,0.00014106820275762628`},{2.1962334797389724`,4.3650554182029575`,0.030303548541137093`,0.006982216802869715`,0.00007989034641895125`,4.909563887726241`,4.785917858478016`,0.006313698593935747`,0.008435258870021976`,0.00007989034641895125`},{2.255025295284807`,4.507331694731078`,0.03096280232164049`,0.01135161953991427`,0.00007495198359767376`,7.296189274151436`,3.1997231189003754`,0.005558793108544652`,0.004439065009892919`,0.00007495198359767376`},{3.391245641593909`,2.179818124433564`,0.014300421854099396`,0.012422412103068138`,0.00009179849368362992`,8.613422552879062`,2.893717202666804`,0.01139391446950836`,0.0051808612699554835`,0.00009179849368362992`},{2.979849118667106`,1.44560591564053`,0.014612773272565327`,0.011231610447480275`,0.00009395998808597482`,9.72166052438573`,4.507330064083695`,0.007399642349368893`,0.0036590585762202275`,0.00009395998808597482`},{1.4271528005484857`,4.531983659998102`,0.015789094378545386`,0.00837375876107373`,0.00008740938833638853`,5.403134305784754`,3.821211664573087`,0.005571246598679358`,0.006825707407031506`,0.00008740938833638853`},{4.308095160219894`,2.9599665905551005`,0.03726156121677038`,0.008268498871797984`,0.00005635520711631662`,9.359286098610681`,2.246017033974116`,0.006345155707154139`,0.005038135865997058`,0.00005635520711631662`},{4.814921564390251`,1.6845565314308066`,0.016824651520304656`,0.011434223130620372`,0.00005220091586155529`,7.574935140112242`,4.495092212641241`,0.009960133044277446`,0.004332095413214537`,0.00005220091586155529`},{2.6176997218528237`,2.045345799033358`,0.02639413294572729`,0.007063496438818283`,0.00007862921069014695`,9.597293422181107`,4.136869561202522`,0.005516279041651674`,0.004577055241717139`,0.00007862921069014695`},{2.3817144988600534`,4.819992176910646`,0.01565878804692078`,0.013475850129921543`,0.000059637198119858164`,9.272375625775478`,2.615572134466057`,0.01103833314363619`,0.008102761570720346`,0.000059637198119858164`},{3.6552584146245435`,3.9985089406701873`,0.020575639376334262`,0.013076049796504865`,0.000040915741337769476`,8.091768727963537`,3.1912415702340162`,0.012743842700060265`,0.0057100082777845375`,0.000040915741337769476`},{4.3964867784207335`,2.7589900026703233`,0.02800763854557428`,0.009348500913631458`,0.00005784081453726595`,6.478759515903601`,4.31931401549907`,0.01594742745602898`,0.004604829650746153`,0.00005784081453726595`},{4.351111271102035`,3.4077086106626586`,0.03605140606313695`,0.007326475409286354`,0.00003550630872206986`,9.98035794376636`,3.648112507031197`,0.011811191186482363`,0.006937988534537566`,0.00003550630872206986`},{3.5336102296011935`,2.3716799866767273`,0.025264773706305088`,0.007106154897859741`,0.000055161409382332906`,9.440594051484961`,4.505584868877075`,0.01743224191737463`,0.005191411929796472`,0.000055161409382332906`},{3.1299455623457115`,3.6761670935858053`,0.013711624536739804`,0.014178742116777103`,0.000055211869795341977`,5.076507137970422`,3.8432886569455755`,0.008903520493942302`,0.007501717901890921`,0.000055211869795341977`},{2.6903130957208834`,2.953292307015974`,0.02648436754612178`,0.009142280141946`,0.00007795851854600945`,6.06738221222615`,4.606281655787372`,0.006715776304114171`,0.00546714794868889`,0.00007795851854600945`},{3.625089606790546`,3.950270801833674`,0.03728765896154164`,0.0066728356716175205`,0.00006649915380543001`,9.667090758047568`,2.1649779002072593`,0.009015419787222943`,0.007984386813379683`,0.00006649915380543001`},{3.7250481522608787`,2.336524551617167`,0.008216002950714778`,0.01679026292360841`,0.00004565526250365458`,8.863468101370557`,4.809386701975686`,0.02148734395598647`,0.0065816242300567075`,0.00004565526250365458`},{1.9024652583135415`,4.108703497836967`,0.03141779940970958`,0.009341808452646101`,0.00009719257137469914`,7.81616190265547`,3.2547478306224447`,0.00926678411288219`,0.002131095683325788`,0.00009719257137469914`},{2.284737610117716`,3.973720798867986`,0.029613133789379895`,0.00886729635524424`,0.00006861933554967205`,5.812539626110274`,4.114260038853255`,0.008316962411123827`,0.00258698939552`,0.00006861933554967205`},{3.408696642908496`,2.6673209251228744`,0.03661380185162268`,0.007915219288753685`,0.00006298240089148044`,9.919021231487953`,3.7131129141629247`,0.005600990500027547`,0.008369244772315793`,0.00006298240089148044`},{2.0796663350015105`,4.826203854127471`,0.029280026652087274`,0.007049349969308796`,0.00007265662730817214`,9.911464702823622`,2.778934140357996`,0.01815923135204415`,0.0023973491505934856`,0.00007265662730817214`},{2.621788630061922`,4.039691969588445`,0.01610350222811118`,0.01410412235009862`,0.00006000437431690365`,8.323804643397274`,2.3853195936335583`,0.009714277529158606`,0.004475144214635587`,0.00006000437431690365`},{4.154654171331198`,2.8413964121239585`,0.037054901795851095`,0.009137556426451452`,0.00006360945785440289`,8.465181114421846`,3.261986290629891`,0.008687588168875687`,0.005322561992855867`,0.00006360945785440289`},{2.3363263496297355`,4.011008384355884`,0.019107154499166813`,0.01267687741734301`,0.00006859572971294422`,5.640123749603429`,4.206488098861133`,0.00939859716450469`,0.0023639811298703725`,0.00006859572971294422`},{3.0149421660592175`,1.8346602200173132`,0.011325558767564477`,0.008924371487147868`,0.00007334865675460967`,7.133459881305727`,4.50579280043028`,0.008877689083285949`,0.008192135974503776`,0.00007334865675460967`},{4.767759684855681`,2.4390472289680423`,0.026426398888206998`,0.010949667396070204`,0.00006443104050005209`,5.296720823556273`,4.190585391755452`,0.008205516852684391`,0.006193921956684497`,0.00006443104050005209`},{2.3462395604261497`,3.7286583565223763`,0.029207990050577337`,0.0074931140714168225`,0.0000625127881781133`,7.307882194202239`,4.722337472940553`,0.007385809606728834`,0.010027653311524252`,0.0000625127881781133`},{4.053881590163644`,3.257523401989898`,0.014815726342390353`,0.014958422339333477`,0.00003907168733330432`,7.896528590253499`,4.5519470627503535`,0.014657417161681903`,0.008497591530188715`,0.00003907168733330432`},{2.4024419022916916`,3.449120813650211`,0.03941844090214695`,0.005383428301635861`,0.00009389208551799822`,6.969072228446176`,3.308905099481996`,0.007741980712542444`,0.002785441029131922`,0.00009389208551799822`}};**

CFFM2(-)

**Table={r_1, K_1, k_1, q_1, w^(2)_1, ,r_3, K_3, k_3, q_3, w^(3)_1}**

**w^(2)_{-1}= w^(3)_{-1}=1, \tilde{w}^(2)_{-1}=1/10.**

**{{2.589591811140913`,3.415662077770686`,0.03496537419378029`,0.005680530460321104`,0.00008345327699795175`,6.5398751319437025`,4.574832981278707`,0.03332712515157758`,0.0031445530429898456`,0.00008345327699795175`},{4.485717596379017`,3.6990410232437476`,0.018431560822567784`,0.009942480878054962`,0.00006548510930044294`,6.912077023405342`,1.9415933274602963`,0.005193677128469555`,0.010957990365690035`,0.00006548510930044294`},{3.1278816756589842`,1.639915725523771`,0.03721436222066671`,0.003951754237714926`,0.00015353438912027039`,9.679789098511801`,2.393875701613905`,0.019637288855376883`,0.004056148089343476`,0.00015353438912027039`},{3.9684349377898096`,3.1036053511511685`,0.021283424163695358`,0.013068154568151968`,0.00012725978428445575`,4.5087685419564725`,2.165736110166719`,0.009710062823193005`,0.0023129223311990285`,0.00012725978428445575`},{3.4216503582138245`,2.9866563171329394`,0.03130990050942686`,0.0037020616183783203`,0.00007656950390010075`,6.131861442862142`,3.695484732914564`,0.01920993427337247`,0.006275870062036171`,0.00007656950390010075`},{1.379173146099168`,1.071844426740399`,0.01838160103143669`,0.002814462697349307`,0.0005843361349272705`,9.006677736370197`,1.968181274048562`,0.016137186425413293`,0.007114744375397917`,0.0005843361349272705`},{3.285961168037222`,3.9207949697994415`,0.01790015548244467`,0.016934948605488002`,0.00012504588439876479`,6.4587711309253155`,1.361575346628623`,0.00589534588947508`,0.003950101694476443`,0.00012504588439876479`},{3.6460687148589948`,1.5751015125448022`,0.02697091022138888`,0.0033293209941545124`,0.00011701361852796562`,8.06000153754242`,4.290603914557414`,0.04264482527149226`,0.0032254185879508103`,0.00011701361852796562`},{2.0323484193178096`,1.1244143244012932`,0.00519616699469249`,0.006171016807644189`,0.0003289122187295295`,4.213362501448156`,4.015253894109251`,0.010972319329611888`,0.012054808481260462`,0.0003289122187295295`},{1.815587994430376`,2.6537986041728727`,0.02973065913365809`,0.004124865501791603`,0.00012824231314032182`,5.484179587820268`,3.796609741030931`,0.013092269984095094`,0.006354211339477583`,0.00012824231314032182`},{3.8617161876485726`,1.4993987839724712`,0.010979224284781215`,0.007042292805436789`,0.00019909342762164796`,3.133237440308333`,3.769756698883591`,0.005209972558697165`,0.01090895248674422`,0.00019909342762164796`},{2.228349044327545`,1.7520982472819888`,0.03311527423768783`,0.002297934275784508`,0.00021747680241069418`,7.196869105918173`,3.005162146847817`,0.02057464745423316`,0.00591239740394392`,0.00021747680241069418`},{1.7474582808618155`,4.482777515796806`,0.01252077099139269`,0.011187130889149376`,0.0001231882806873154`,5.755894717195165`,2.351898326548607`,0.015282336438301153`,0.0033986599109034066`,0.0001231882806873154`},{2.8671585021353305`,2.156048609800443`,0.02641534413597653`,0.0043556294790928485`,0.00010215963753639726`,9.637918428567197`,2.1512216483098143`,0.016890654848242148`,0.004119064422071016`,0.00010215963753639726`},{3.6851347958322886`,1.2366882775146681`,0.03476724891911283`,0.002614904926373944`,0.00022977521662696549`,3.7218127814061717`,4.775257681698269`,0.015119320803709334`,0.007722713262970297`,0.00022977521662696549`},{4.899908115790179`,1.2752946864774755`,0.031671960998106874`,0.002288087981009424`,0.00013963659600943912`,9.638210913083384`,2.1636550601170876`,0.018398903035019336`,0.007997389499441714`,0.00013963659600943912`},{1.5866444478572754`,4.732120454151721`,0.04530109013916436`,0.006402661658939147`,0.0001849442049913772`,9.286589613830571`,1.346322125413053`,0.012467464705195087`,0.00299972698393847`,0.0001849442049913772`},{2.3723276291293915`,1.1580462778103522`,0.00796587973060419`,0.0031303259013329907`,0.00017412355771582858`,8.997764950062447`,2.5493097935105995`,0.01528791464310144`,0.012369738775870442`,0.00017412355771582858`},{4.41472399009293`,1.2530937230396937`,0.04095575145423698`,0.005927301470090917`,0.00019908172480490862`,4.8865270103189715`,3.0218520496240258`,0.0073039370044618995`,0.0055481531965531385`,0.00019908172480490862`},{1.5152298982772203`,4.594477199998162`,0.020982304314467806`,0.007613695338064785`,0.00017305917174459872`,6.001798007800257`,1.9149411136548045`,0.011281851079749168`,0.0033094074921395586`,0.00017305917174459872`},{1.3594619511002106`,2.8708663628354953`,0.012151239093767217`,0.003807137585981498`,0.00013278119520002043`,7.131543227411786`,3.353024645242967`,0.009767797022219186`,0.019627627669456967`,0.00013278119520002043`},{1.8021785120559723`,2.3126836637359807`,0.00581745382269528`,0.017717363148382476`,0.00016417176608291375`,9.17256399906723`,2.6943876169036693`,0.031213541155161353`,0.002131833540176674`,0.00016417176608291375`},{1.842660834361764`,3.7862845658785327`,0.03487391770214443`,0.0020147404592559676`,0.00009660563664345891`,5.598860973655816`,4.4622235488962385`,0.012310023455383373`,0.01505963659828486`,0.00009660563664345891`},{2.488653234988589`,3.451384058591419`,0.039097247325648066`,0.0025170771237106836`,0.0000753027938965123`,8.84280489829893`,3.620671812705356`,0.027170699728583297`,0.007699393302396097`,0.0000753027938965123`},{1.7989586165708653`,1.011833395716578`,0.005433728182763303`,0.017316455125523118`,0.00043260719127286473`,3.9245915483767373`,4.5407224722105015`,0.0220756869423879`,0.0021907402769900074`,0.00043260719127286473`},{2.5189430369010175`,1.5125139706399997`,0.006220244913811027`,0.019099208938965256`,0.00023943712649624653`,6.404387194504185`,2.2731349859823116`,0.015609023532789673`,0.0038662153690908266`,0.00023943712649624653`},{3.927759274955129`,3.0899228566789887`,0.04311964936675894`,0.005539452530583765`,0.00007394763055712625`,3.994137913194381`,4.2871509367542995`,0.005220987877599892`,0.009576808340582544`,0.00007394763055712625`},{4.601080032859458`,2.7386432675038694`,0.04866773233471357`,0.004762327435048567`,0.00010050454051656137`,3.9888510172238263`,3.626896927815367`,0.01533335139999055`,0.003853202457893834`,0.00010050454051656137`},{2.2464392928874606`,4.5763506986451175`,0.030832341012034373`,0.005038423269186097`,0.00010036235210448131`,3.68590161007228`,3.24292116521765`,0.006158008031339701`,0.008594013417133731`,0.00010036235210448131`},{1.7804096826563995`,4.834372514021803`,0.029035755776609523`,0.003333836911965423`,0.00009146502570452357`,4.165559476082114`,4.685866854603434`,0.007389896659135253`,0.013350058238471036`,0.00009146502570452357`},{3.3970172033230295`,4.482155624061308`,0.014449188999987254`,0.014454802332220892`,0.00004640986697625497`,8.807522682588747`,3.5404702102921917`,0.042684248051912024`,0.0027009337465888766`,0.00004640986697625497`},{1.5982726664793532`,2.90676897453457`,0.030619745883483762`,0.003400839873269494`,0.00019286283162875894`,5.240628858867533`,3.733123479875742`,0.0067899704904426095`,0.016588598065531448`,0.00019286283162875894`},{4.082131013549285`,1.0063162306254547`,0.0408827698149037`,0.008020815775256582`,0.00018232155316974638`,5.7200223223307365`,3.093558445105254`,0.007372630704429693`,0.002551643445983267`,0.00018232155316974638`},{1.3472712997177396`,3.9696161878178797`,0.005832485172135901`,0.011909515683854628`,0.00016098191353637083`,5.155619303703684`,3.1764872740936623`,0.005872881460968912`,0.01740345036210802`,0.00016098191353637083`},{3.423084056175112`,3.0102203723819585`,0.03812722822844608`,0.006429310215475707`,0.00009355317589311422`,4.994398219927362`,3.07129123922875`,0.01176329499310854`,0.0037458750112443207`,0.00009355317589311422`},{4.650421525045175`,3.795586263694907`,0.025681396324034753`,0.010200220447962559`,0.000049456701335389526`,4.32707575536317`,4.7130214926075045`,0.023838760093973108`,0.002452737686761637`,0.000049456701335389526`},{2.9833530655567158`,3.072624440270544`,0.04000079375701429`,0.008071943049281927`,0.00012563888789471562`,4.543380184667665`,3.506721053038401`,0.018318294495573248`,0.0020791204864759154`,0.00012563888789471562`},{2.778485870085812`,3.850481734831245`,0.00980284699722455`,0.015210303641027378`,0.0000879759400608329`,7.356877802345842`,2.2677286824714944`,0.024568229028087918`,0.002241050497779968`,0.0000879759400608329`},{1.0902857640673194`,1.283763766730436`,0.04776637667425436`,0.002573707642748148`,0.0007785009564591305`,3.691709352623471`,4.528518329454364`,0.010899334206429225`,0.005062634568047623`,0.0007785009564591305`},{4.793812146337198`,4.475482159480262`,0.011733201375878098`,0.01712341492667636`,0.000032608214744329466`,6.521070249962522`,3.4885630982825226`,0.016567330233028643`,0.008734228382515773`,0.000032608214744329466`},{1.3832213418700192`,4.84138574838478`,0.019925755187741936`,0.008614407203842184`,0.00009792948910958429`,6.780948252584043`,4.003255852481852`,0.03075244365340657`,0.002421985991199395`,0.00009792948910958429`},{1.4455022498514367`,2.5391234742539446`,0.02392788675909059`,0.0022973057741143034`,0.0001564947237448289`,8.97081960200369`,3.5261142096063605`,0.015970072222603333`,0.014463177059265676`,0.0001564947237448289`},{1.3382998460902655`,3.4233749976271133`,0.0213838106823923`,0.006016809142680057`,0.0002858992161876078`,2.492832808946112`,3.714554150764301`,0.005803379566326275`,0.005487307637595411`,0.0002858992161876078`},{4.452523260933805`,1.5827609665622786`,0.006695187647233813`,0.01191189458809188`,0.00010966305398945209`,4.977936508574658`,4.43886662764289`,0.02848718967497474`,0.005297172468602576`,0.00010966305398945209`},{4.1984141204291205`,3.195014245525588`,0.04177571424485681`,0.009088147313415309`,0.00009507849764750238`,3.866355149593849`,2.794047673736287`,0.007057580805418073`,0.00395180928626181`,0.00009507849764750238`},{4.343935625081391`,1.3314299857889091`,0.029410746343635982`,0.003032172138474506`,0.00014323030354041724`,9.425306779497898`,2.0519862983221575`,0.012412255916334311`,0.01110354173263222`,0.00014323030354041724`},{1.6485158835103908`,1.4356725430781365`,0.049285466509261314`,0.004833212512072773`,0.0004980018981756505`,3.4525168382091422`,3.5548250589460766`,0.005513790910084547`,0.0049894851369307325`,0.0004980018981756505`},{1.242308941931623`,1.7612274029081458`,0.03210135769383361`,0.00485914874107914`,0.00047987401733181755`,8.326827049052085`,2.0428380166718565`,0.018971249780016228`,0.0025419669587719816`,0.00047987401733181755`},{1.2541595159508914`,3.0920474445172825`,0.035506521710756245`,0.005370513445923132`,0.00024040801898944707`,5.966003405586537`,2.1179062312958195`,0.006919471270268547`,0.0046601398975622925`,0.00024040801898944707`},{1.6166474675106475`,3.900433801103836`,0.011682214831664803`,0.008323034595621497`,0.0001518590836745617`,6.133588861996309`,2.288158204774609`,0.005228749849075141`,0.012601202346862198`,0.0001518590836745617`}};**

CFFM3(-)

**Table={r_1, K_1, k_1, q_1, w^(3)_1, ,r_3, K_3, k_3, q_3, w^(3)_1}**

**w^(3)_{-1}=1, \tilde{w}^(3)_{-1}=1/10.**

{{4.0567553634030284`,1.878984174437579`,0.028050780642697046`,0.003997161839287111`,0.00009914512717954633`,8.825294231292045`,2.980727341339855`,0.022600326350836475`,0.009841905102594275`,0.00009914512717954633`},{1.0019662341732039`,1.3039713056998217`,0.04424271420063032`,0.002800479168317941`,0.0005871805651309896`,8.723756867043157`,4.797504638308936`,0.041756629421523306`,0.006164535191160066`,0.0005871805651309896`},{1.587292470704707`,4.536125969658152`,0.019794484565027172`,0.004108860121427537`,0.00007555872680597964`,8.992363684279177`,4.92995321293833`,0.04376363396622253`,0.008324030293098372`,0.00007555872680597964`},{1.1343657842223225`,3.4929445947595097`,0.008585062420281939`,0.005912236905325559`,0.00016236605383586952`,6.093085352022088`,3.2828717894541537`,0.007344408919222306`,0.019338308969022864`,0.00016236605383586952`},{4.57188968468653`,1.6634943495906072`,0.046733604076368285`,0.00313528017736793`,0.00011636106065504494`,8.358893034162918`,3.9226093019019093`,0.03200114441065692`,0.008628614675914342`,0.00011636106065504494`},{1.4939496812914008`,2.42578244232614`,0.03277633685160557`,0.006756261603139014`,0.0002935966742585558`,4.076518948658929`,3.1362899109515343`,0.006479821591719642`,0.005663076263380237`,0.0002935966742585558`},{2.8341581301165224`,1.1479662506394908`,0.032531921167068725`,0.0027827798610973904`,0.0003332165288033662`,4.337814351818128`,3.9219852037230627`,0.011789145596621906`,0.01145050383538106`,0.0003332165288033662`},{1.0851593878773036`,1.0850319970497022`,0.010660563596989935`,0.017305604511378702`,0.0006936584664090088`,2.753054295335808`,4.654418459040021`,0.009743689522453212`,0.0026267799655432036`,0.0006936584664090088`},{3.1856907999424253`,4.294909738522062`,0.027554602229721212`,0.007708171664259092`,0.00010026582900173595`,8.238905910272006`,1.5550381484610885`,0.014566051453733145`,0.0035681624730213093`,0.00010026582900173595`},{1.812896891097406`,3.365554273608522`,0.023233703351781057`,0.003961712792432914`,0.00011002764654842803`,7.405418479740209`,3.1234288396358414`,0.019278600921860872`,0.007321947051844788`,0.00011002764654842803`},{3.361134396072643`,4.3844052969703675`,0.04129952270388536`,0.002651360584477312`,0.000056813295367912596`,9.249399804299369`,3.42209692267783`,0.02476003244868076`,0.013482843076983383`,0.000056813295367912596`},{1.3680313550999346`,1.2744625219427395`,0.0318476179539966`,0.005918603094527308`,0.00030118249161033175`,9.611956140851596`,4.001343027626501`,0.035426505692551596`,0.0022991242780439243`,0.00030118249161033175`},{1.5707263141522123`,3.7348850601421546`,0.006670185019678095`,0.004368913507604131`,0.00006700506803924392`,9.847437606893081`,4.5212263434301505`,0.03285592498908496`,0.0162196645078817`,0.00006700506803924392`},{4.771748767334697`,3.3426396992599923`,0.022155666295228048`,0.016229356871652296`,0.0000730120688412412`,8.60019153280654`,1.2482243096884265`,0.007256028044782287`,0.0027840358401207445`,0.0000730120688412412`},{4.776111952287646`,1.1020156042316005`,0.021158497492960386`,0.0077282311867656085`,0.00011184139598443372`,9.66326954839446`,3.161578288408964`,0.03431221284263161`,0.0025925954040557017`,0.00011184139598443372`},{1.815894167545924`,2.4345231117089883`,0.01834358476962019`,0.005894470108955718`,0.0001568751350226414`,4.915952515386474`,3.674461608464635`,0.008810141897357342`,0.010104429112049479`,0.0001568751350226414`},{2.8007827511461114`,1.5301732720872208`,0.04626919877249143`,0.0027053278266067106`,0.00021034688902411228`,7.6643500550812185`,4.566278541552457`,0.04109641435939057`,0.005408563873670436`,0.00021034688902411228`},{3.5426878018837336`,2.739059203100923`,0.044338654911525735`,0.0052068317271437466`,0.00013714019688772748`,3.617599306412293`,3.4619401130788123`,0.012179131580018578`,0.004343042988097078`,0.00013714019688772748`},{3.472526911364535`,2.650730846088126`,0.03869836326062066`,0.00590669855052214`,0.00014714443223450696`,3.9836841415759423`,2.904593492919468`,0.005946359741182745`,0.009287828586477274`,0.00014714443223450696`},{2.5909212820640795`,1.8596620404469144`,0.04655609290211467`,0.015637003357888025`,0.00023012593561613478`,2.888062900782421`,4.782574210029125`,0.0051500110215405015`,0.002136258016012131`,0.00023012593561613478`},{2.349926213209957`,3.119614576309562`,0.04014749833842364`,0.004873811286385064`,0.00014252390283178663`,4.9910135654165995`,4.023494174377054`,0.02562640948846147`,0.0028417454738119234`,0.00014252390283178663`},{2.6546719061839976`,3.3396211434840293`,0.028483591126247557`,0.004033366149079079`,0.00006295512406012346`,8.835745073670225`,3.9994797098134924`,0.03369808360598825`,0.006339671130028115`,0.00006295512406012346`},{1.888967665584941`,2.845246380873082`,0.0398144212422403`,0.004107234800117349`,0.0002015890991537018`,7.533677897537581`,2.2089951154748393`,0.014296725935713815`,0.006581610987360462`,0.0002015890991537018`},{1.1489220909279467`,4.018495775644467`,0.012036196536259085`,0.012839986927838245`,0.0003742647686540562`,4.0024328482684695`,2.1610013940801123`,0.009373544608152207`,0.0028848794090720505`,0.0003742647686540562`},{1.4519271077550568`,1.0129263822294226`,0.009232244014764503`,0.009324300033266005`,0.0005719878625292764`,7.225798295256643`,1.8283391308535064`,0.0056699475768293844`,0.012161109455237157`,0.0005719878625292764`},{3.4610073033533384`,1.265637824510402`,0.03163256665637221`,0.0035870002223958065`,0.0002649062370091422`,5.17318101236949`,3.2516798093939245`,0.00952477413318844`,0.014415239849068052`,0.0002649062370091422`},{1.1069325335970062`,4.441363709311115`,0.015605556062369352`,0.003801797130162491`,0.00010022875013270872`,7.972529329814494`,4.259867555515755`,0.0330244489039829`,0.007859039386093936`,0.00010022875013270872`},{1.084444073266754`,2.2349018544368935`,0.02130683029565527`,0.002041698597568753`,0.0002555942855352485`,8.021472999924676`,4.450990646391578`,0.027188347601428506`,0.015445650819674441`,0.0002555942855352485`},{1.567970734837333`,3.7295213512030134`,0.024151702646762252`,0.015391336598752869`,0.00029420593904179904`,6.94246618796126`,1.2696388877637181`,0.0054023478202074315`,0.003004436610441665`,0.00029420593904179904`},{3.78888065539694`,4.151163856821237`,0.008564929486669988`,0.0068669131735492675`,0.00004819029865392368`,9.949680922416924`,1.9045768841414095`,0.01280843602959466`,0.012530555573606644`,0.00004819029865392368`},{1.5022204492540805`,3.762425813509825`,0.04279455925978977`,0.012932155490616395`,0.00042764591508766075`,4.0248064765809115`,1.8809196805344444`,0.005130427090381841`,0.002669364716574203`,0.00042764591508766075`},{1.4482706912050656`,1.7500392115432302`,0.01321275655029789`,0.012387074838330823`,0.0005362336753779169`,7.085563613282577`,1.2889719632121563`,0.00628984972017891`,0.004323374865164542`,0.0005362336753779169`},{1.9299132146598579`,1.2576954768774318`,0.007930133775363925`,0.010688324780106145`,0.00036120976927013493`,5.288206396811921`,2.4706605046654353`,0.010714947580763516`,0.007055478678446941`,0.00036120976927013493`},{2.9248483020925082`,4.764196977400977`,0.005970581174107946`,0.013522195450096086`,0.00003967942506697634`,4.185804947600438`,4.981481395773729`,0.012603598849266755`,0.009795459865148334`,0.00003967942506697634`},{3.2208925810611513`,1.6584752288382276`,0.0072082755435106255`,0.013293232065251098`,0.00017737555451219984`,4.762691825987552`,2.7026451604291726`,0.005664963129438032`,0.010979336506985828`,0.00017737555451219984`},{1.2738774053487045`,1.8420645664055986`,0.021259537722845702`,0.003587569739044151`,0.00024206140273422077`,7.3165689771477975`,4.217371001771877`,0.019054445101653585`,0.012983529432312357`,0.00024206140273422077`},{1.4389791958263318`,4.890370854436175`,0.00610133271688372`,0.011449145719222752`,0.00009799192712695243`,9.349191145424523`,2.0547061010886534`,0.00998470518926093`,0.015608746026378451`,0.00009799192712695243`},{3.2962842449014564`,2.313533029280042`,0.014203356680029249`,0.003763454061093057`,0.00006830898679326082`,9.455061776890417`,3.2454354383533017`,0.028004391072069548`,0.007923296863676065`,0.00006830898679326082`},{1.9849335404593713`,4.774080964085573`,0.0467847474619834`,0.004516211508933127`,0.00006174222463640631`,8.204253737131683`,4.478865874796123`,0.028079818453563582`,0.0030254935715729667`,0.00006174222463640631`},{3.7542928393575377`,1.946562381196907`,0.03727310737241281`,0.010553957424455092`,0.00023801156061834818`,3.220857815950179`,2.6656544414431664`,0.006171902633157263`,0.002448218942473889`,0.00023801156061834818`},{4.614938080310405`,4.132635946148671`,0.013466501341634456`,0.019432074127531448`,0.00006135478399556097`,8.080815276054171`,1.287518497420117`,0.01121113090676567`,0.0021251582008918017`,0.00006135478399556097`},{1.621105211272261`,1.488384860828222`,0.012269385759746995`,0.004263858878754058`,0.00017514887823707548`,8.114127682292434`,3.5500157422070036`,0.018052134621019615`,0.010867771426801356`,0.00017514887823707548`},{1.5219572752960104`,2.5649394656276625`,0.04747638898520007`,0.003445206533874208`,0.0003074483593719286`,4.476039067274682`,4.291069743231934`,0.018949655081681782`,0.006766460147853502`,0.0003074483593719286`},{2.7913565134507543`,1.8591041420195111`,0.008923842268976363`,0.01865551706024338`,0.00021676164381487359`,3.8511635106779156`,2.817009575968293`,0.008285632880161771`,0.0046642348067180775`,0.00021676164381487359`},{1.8249737482885928`,1.161967905170191`,0.03202309115083839`,0.008830025044319009`,0.0008488407843904313`,2.6413337112558306`,3.2816830372682126`,0.005761002104322392`,0.0027062209698402173`,0.0008488407843904313`},{2.855159812818356`,1.5534828476542515`,0.011441626060558235`,0.012983690723366002`,0.00011460677675037513`,6.4125853904782595`,4.227886282986587`,0.026920857359803985`,0.002525237423046528`,0.00011460677675037513`},{2.397015404076619`,2.1846684076596246`,0.012426793232502002`,0.00628961945845519`,0.000123860471638885`,4.390189368280021`,4.746332930249268`,0.008371111420518343`,0.018614980748600757`,0.000123860471638885`},{1.3636294276773322`,3.388748283488451`,0.032320701800499965`,0.003543512990176686`,0.00018191129080665945`,8.252797884939966`,1.9709151703116534`,0.0079302669135633`,0.012788768947257854`,0.00018191129080665945`},{1.8422124096332473`,2.594600756993528`,0.007577260160801694`,0.009984400867544537`,0.00013542222903900982`,8.674421955040255`,3.222957988127315`,0.03545271495468458`,0.005542054162022161`,0.00013542222903900982`},{1.9532638278255323`,1.8854524746102834`,0.037605000551325665`,0.004055328081819718`,0.00030285337770433445`,6.1416548184352315`,2.278914824305411`,0.012065214292022355`,0.006486270190268498`,0.00030285337770433445`}};
